# Bacterial defense mechanisms against bacteriophages: an evolutionary arms race

**DOI:** 10.1007/s00203-026-04785-x

**Published:** 2026-02-23

**Authors:** Rafwana Ibrahim, Jesil Mathew Aranjani

**Affiliations:** Department of Pharmaceutical Biotechnology, Manipal College of Pharmaceutical Sciences, Manipal Academy of Higher Education, Manipal, India

**Keywords:** Bacterial immunity, bacteriophage resistance, bacteriophage counter-defense, CRISPR‒Cas, restriction-modification, abortive infection

## Abstract

Bacteria and bacteriophages are in a co-evolutionary arms race, developing intricate bacterial defense mechanisms that enable phage resistance and counterstrategies. Bacteria evolve diverse defense mechanisms to inhibit each stage of the phage infection cycle.Surface-based defenses prevent phage adsorption and infection, including receptor modifications, capsule production, and biofilm formation. Intracellular systems such as restriction-modification (R-M) and abortive infection (Abi) mechanisms degrade phage DNA or sacrifice infected cells to protect the population. Adaptive immunity, particularly through CRISPR-Cas systems, enables bacteria to recognize and neutralize recurring phage attacks. Phages counter these defenses through anti-CRISPR proteins, receptor mimicry, and depolymerization, which degrade capsules and biofilm matrices. These dynamic interactions shape microbial ecosystems, offering insights for the development of novel antimicrobial strategies. Emerging approaches, including engineered phages and combination therapies, hold promise for addressing bacterial resistance. Understanding these bacterial-phage dynamics is critical for advancing phage therapy as a powerful tool against multidrug-resistant bacterial infections. This review aims to systematically examine and integrate current knowledge on bacterial antiphage defense systems and the evolutionary adaptations employed by bacteriophages to overcome these barriers.

## Introduction

Bacteriophages, or, phages, are viruses that infect and multiply within bacteria, which are one of the most prevalent organisms on earth and are integral to microbial ecology and evolution. With an estimated 10³¹ phage particles globally, they outnumber bacteria by approximately 10:1, influencing bacterial populations in virtually every environment, from the human microbiome to extreme ecological niches ranging from deep-sea vents to polar ice caps (Breitbart and Rohwer 2005; Suttle 2007). Phages drive nutrient cycling, horizontal gene transfer, and the regulation of bacterial community dynamics, thereby shaping microbial population structure and sustaining co-evolutionary interactions between bacterial hosts and their viral predators.(Labrie et al. 2010).

The relationship between bacteria and phages is characterized by perpetual evolutionary arms race. As phages evolve mechanisms to infect and exploit bacterial cells, bacteria have evolved these diverse defense systems to resist phage infection. These adaptations ensure bacterial survival and promote phage diversity, leading to global co-evolutionary dynamics that impact microbial ecosystems (Samson et al. 2013; van Houte et al. 2016). Understanding these defense systems is critical for comprehending microbial life and applying this knowledge in medical, industrial, and environmental contexts.

Initial bacterial defenses are deployed at the adsorption stage of phage infection, where phages recognize specific receptors on bacterial surfaces. To prevent this, bacteria can modify or mask their receptors, employ decoy molecules, or form biofilms to create a physical barriers. Upon phage genome injection, intracellular defense mechanisms are activated. Restriction modification (R-M) systems recognize and cleave foreign DNA while sparing the host genome through methylation. Other systems, such as abortive infection (Abi) mechanisms, act as a population-level defense mechanism by inducing programmed cell death in infected cells to prevent phage replication and subsequent spread (Oechslin 2018).

This co-evolutionary dynamic has led to the development of diverse bacterial defense mechanisms against phage predation. These mechanisms span multiple levels, including the prevention of phage adsorption through surface-based modifications, degradation of phage genetic material via restriction-modification systems, and adaptive immunity via CRISPR‒Cas systems.

Moreover, bacterial defenses are increasingly recognized as valuable molecular biology and genetic engineering tools. CRISPR‒Cas has revolutionized genome editing, allowing precise modifications in diverse organisms (Hryhorowicz et al., 2023). Similarly, restriction-modification systems have been exploited for cloning and recombinant DNA technologies (Hsu et al., 2014). Phages, in turn, have evolved strategies such as anti-CRISPR proteins, receptor mimicry, and modified genomes to escape bacterial defenses. This interplay results in a dynamic co-evolutionary relationship that shapes microbial ecosystem structure, regulates bacterial population dynamics, and drives genetic diversity through horizontal gene transfer(León & Bast\’\ias, 2015; Oechslin, 2018).

The study of these defense systems is not merely of academic interest but has practical implications. These findings have profound implications for biotechnology and medicine, particularly in the context of phage therapy, which is emerging as a potential solution to combat the increasing threat of multidrug-resistant bacterial infections. Although phage therapy offers several advantages, including specificity and self-limiting replication, bacterial defense mechanisms often pose significant challenges to its efficacy (Paoli et al., 2025). Understanding these mechanisms helps in modifying engineered phages capable of bypassing bacterial defenses and enhancing therapeutic outcomes (León & Bast\’\ias, 2015; Oechslin, 2018). Notably, receptor modifications and CRISPR-mediated immunity can render therapeutic phages ineffective, necessitating the development of engineered phages or combination strategies to overcome bacterial resistance. Additionally, bacterial defense systems, such as R-M and CRISPR‒Cas, have become indispensable tools in biotechnology, facilitating advances in genetic engineering, synthetic biology, and molecular cloning (Barrangou & Marraffini, 2014; R. J. Roberts et al., 2015).

This review focuses on the intricate interplay between bacteria and phages, categorizing bacterial defense mechanisms into surface-level and intracellular strategies, and adaptive immune responses. We explore the molecular underpinnings, evolutionary significance, and applications of these systems, offering insights into their roles in shaping microbial ecosystems and their translational potential in medicine and industry. Elucidation of these interactions contributes to a comprehensive framework for understanding bacterial antiphage immunity and its translational relevance. This review categorizes bacterial defenses into surface-level, intracellular, and adaptive immune systems, while systematically highlighting the corresponding phage counterstrategies that sustain co-evolutionary dynamics and shape the organization of subsequent sections.

## Bacterial defense mechanisms

The ongoing battle between bacteria and bacteriophages is a defining feature of the microbial ecosystem. To survive the relentless pressure exerted by phages, bacteria have evolved diverse defense mechanisms that function at various stages of the infection. These strategies protect individual cells and often benefit bacterial populations, highlighting the complex interplay between host survival and community resilience.

Phage infection typically begins with the adsorption of the virus to specific receptors on the bacterial surface, followed by the injection of genetic material into the host cell. Each stage of this process represents an opportunity for the bacteria to deploy countermeasures. Bacterial defense mechanisms are as diverse as they are effective, ranging from preventing phage adsorption to degrading foreign DNA and even inducing self-sacrifice to protect the broader population, .

**Fig. 1:**
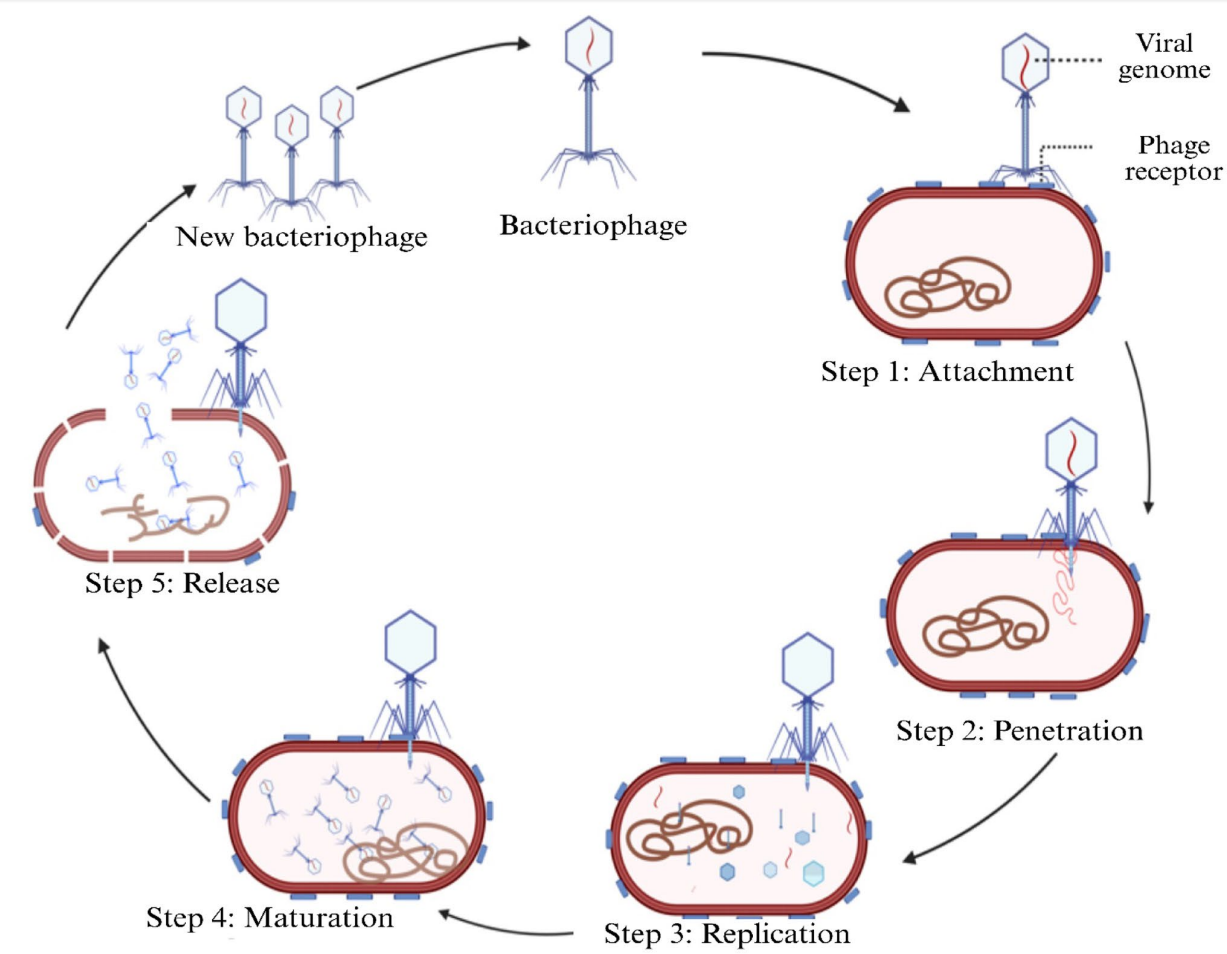
Life cycle of lytic bacteriophage.Created in BioRender. Aranjani, J. (2026) https://BioRender.com/tc69wi2

An understanding of the lytic bacteriophage infection process provides essential context for interpreting bacterial defense mechanisms. (Fig. 1). Phage infection is a meticulously coordinated series of steps, each presenting a potential vulnerability for the bacterium and an opportunity to mount a defense (Gordillo Altamirano and Barr 2019).

Phage infection begins with adsorption and host recognition, in which phages attach to specific receptors on the host bacterial surface. These receptors are often vital bacterial components, such as lipopolysaccharides (LPS), teichoic acids, or pili, ensuring precise phage targeting (Sahu et al. 2023; Selvaraj and Singh 2020; Stone et al. 2019). However, this specificity also creates a potential weakness for bacterial defense. Bacteria can adapt by modifying or masking these receptors, secreting extracellular polysaccharides, and forming protective biofilms to obstruct phage attachment. Such strategies are often observed in bacterial populations exposed to high phage predation (Hyman and Abedon 2010; Pires et al. 2017; Y. Wang et al. 2023a, b, c).

Following successful adsorption, the second stage is the DNA injection phase, in which the phages inject their genetic material into the host using specialized tail structures or enzymes that pierce the bacterial cell wall and membrane. Once inside, the phage genetic material gains access to the bacterial cytoplasm, initiating infection. At this stage, some bacteria limit phage genome entry by reinforcing or remodeling their cell envelope, through peptidoglycan thickening, altered cross-linking, or modifications of membrane-associated structures that restrict DNA translocation across the cell envelope (Hyman and Abedon 2010; Labrie et al. 2010). In parallel, bacteria deploy intracellular surveillance and defense systems that detect and neutralize incoming foreign DNA before it can be established within the host cell.(Brüssow et al. 2004; Dicks and Vermeulen 2024; Young 2005).

Once inside the host, phages shift to the replication phase, where they reprogram and manipulate the bacterial cellular machinery to prioritize the production of phage components. Phages often suppress host defenses, degrade bacterial DNA, and redirect resources to construct new virions (Teklemariam et al. 2023). This stage is a critical vulnerability for bacteria, as the host effectively loses control over cellular processes. In response, bacteria have evolved sophisticated countermeasures, such as restriction-modification systems, which recognize and degrade phage DNA, and CRISPR‒Cas systems, which offer adaptive immunity against previously encountered phages. Additionally, some bacteria employ abortive infection strategies, triggering programmed cell death to protect the broader population from infection (Barrangou and Marraffini 2014; A. G. Johnson and Kranzusch 2022; Smith et al. 2023; Tal et al. 2022).

During assembly phase, phages package their genetic material into newly synthesized capsids and construct other structural components (Rao et al. 2023; Roos et al. 2007). While this stage is less immediately harmful to the bacterial cell, it drains host resources, potentially weakening its survival ability. Bacteria can respond by activating stress responses that disrupt phage assembly or initiating defense mechanisms that interfere with phage maturation (Fernández et al. 2018; Hasan and Ahn 2022).

The final stage involves cell lysis and phage release, where the bacterial cell wall is destructed by phage-encoded enzymes such as endolysins and holins, releasing newly formed virions to infect nearby cells (Dennehy and Abedon 2021; Fernandes and São-José 2018; Gao et al. 2020; Young 2005). This stage is particularly catastrophic, killing the host and amplifying the threat to the neighboring bacterial populations. Some bacteria counteract this by utilizing phage-inducible chromosomal islands (PICIs), which inhibit cell lysis or produce extracellular inhibitors to neutralize phages after their release (Taati Moghadam et al. 2020; Teklemariam et al. 2023).Therefore, each stage of the phage infection cycle represents a critical interaction between the predator and prey, in which the bacteria must either adapt or succumb. These interactions have driven the evolution of diverse and intricate bacterial defense mechanisms, many of which are tailored to specific stages of infection. This interplay has significant implications for microbial ecology, evolutionary biology, and applied phage therapeutics. (Hyman and Abedon 2010; Labrie et al. 2010).

**Fig. 2:**
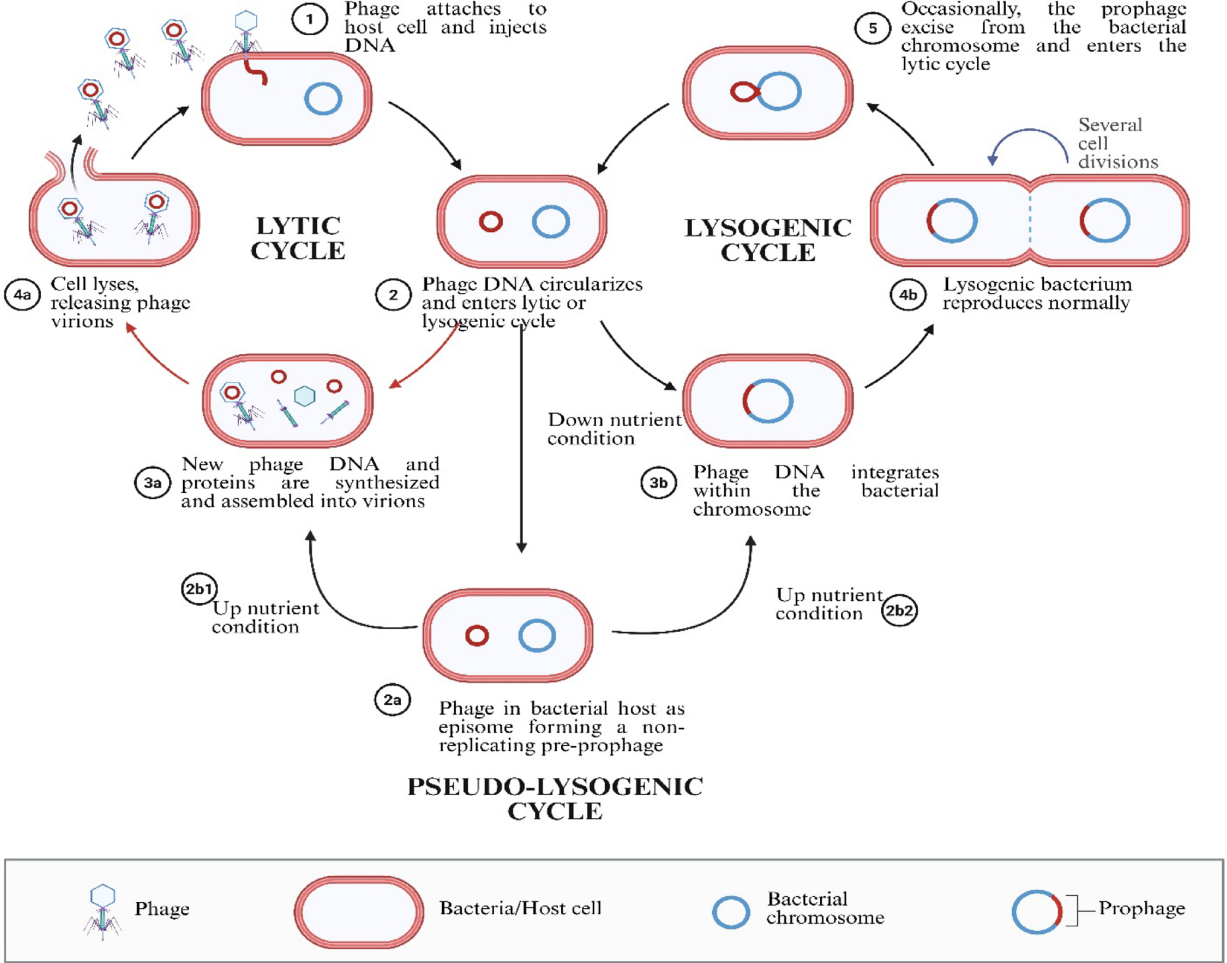
Schematic representation of bacteriophage infection pathways illustrating the lytic, lysogenic, and pseudo-lysogenic cycles.Created in BioRender. Aranjani, J. (2026) https://BioRender.com/tc69wi2

Bacteriophage infection follows multiple pathways beyond canonical lysis, including lysogeny, chronic infection, and pseudo-lysogeny. In addition to the canonical lytic pathway outlined above, phages can follow alternative developmental routes that are strongly influenced by the host’s physiological status and environmental conditions. These include lysogeny, in which the phage genome integrates into the bacterial chromosome and is vertically transmitted during cell division; chronic infection, characterized by the sustained production and release of progeny virions without overt host lysis; and pseudo-lysogeny, a transient state in which the phage genome is maintained episomally under conditions of nutrient limitation or cellular stress until a transition to either lytic or lysogenic growth becomes favorable(Ngene and Umar 2025; Zhang et al. 2022). The coexistence of these distinct infection strategies highlights the conditional and dynamic nature of phage–host interactions and broadens the range of stages at which bacteria can deploy effective defensive countermeasures (Fig. 2).

**Fig. 3:**
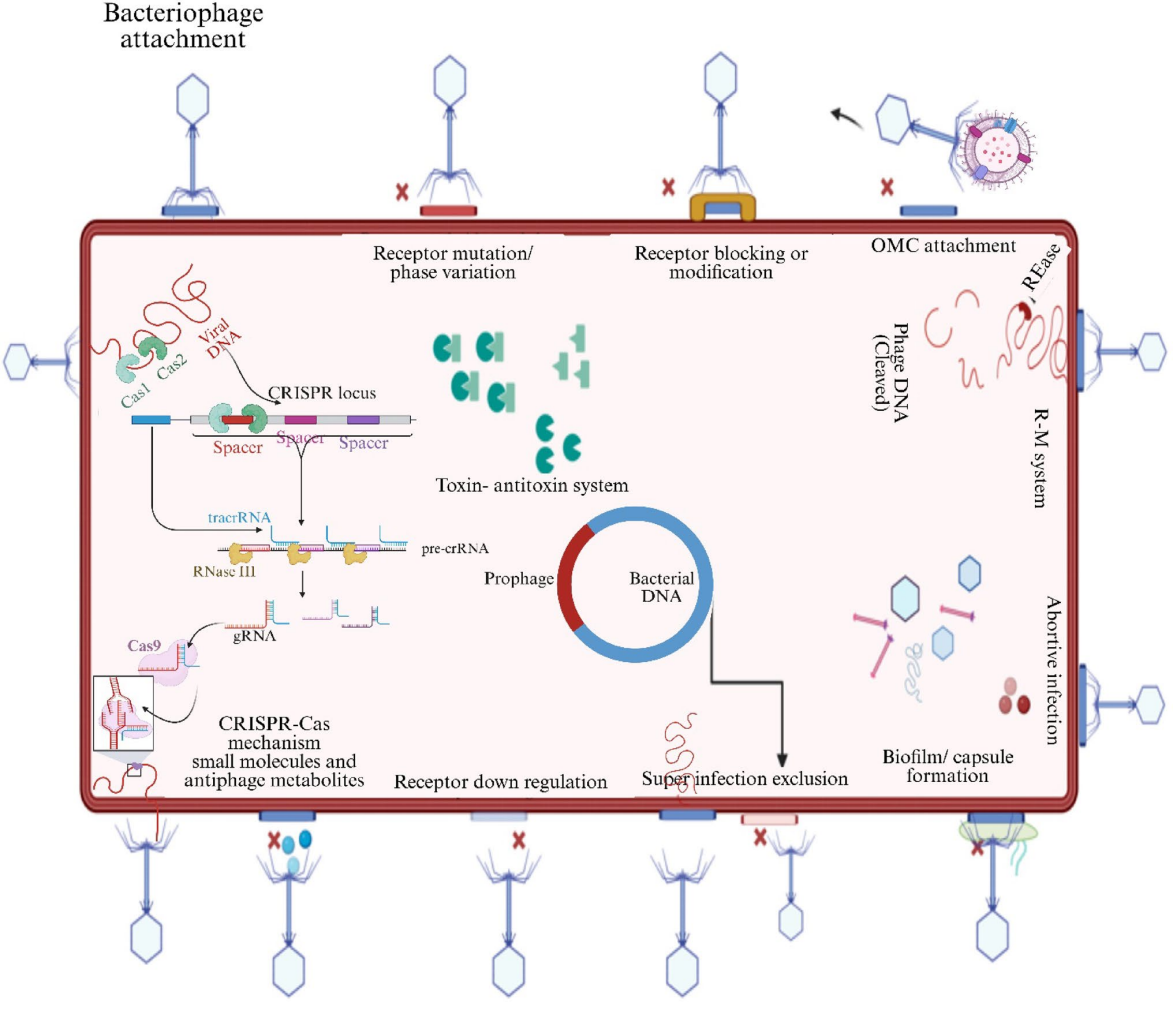
Bacterial defense mechanisms against bacteriophages. The schematic illustrates various strategies employed by bacteria to counteract bacteriophage infections. These include receptor-based resistance mechanisms such as receptor mutation/phase variation, receptor blocking/modification, and receptor downregulation. Surface-associated defenses include outer membrane vesicle (OMV) attachment and biofilm or capsule formation. Intracellular defense systems involve restriction-modification (RM) systems, CRISPR‒Cas mechanisms, toxin‒antitoxin systems, abortive infection pathways, and superinfection exclusion. Additionally, bacteria utilize small molecules and an-tiphage metabolites to disrupt phage replication. These multilayered defense strategies collectively enhance bacterial survival against phage predation Created in BioRender. Aranjani, J. (2026) https://BioRender.com/tc69wi2

The following sections, detail about bacterial defense mechanisms, categorizing them based on their role in combating specific stages of the phage infection process (Fig. 3). Broadly, bacterial defense strategies can be categorized into three major types: surface-based defenses, intracellular defenses, and adaptive immunity via CRISPR‒Cas systems (M. R. J. Clokie et al. 2011; Madsen et al. 2012). Surface-based defenses involve modifications of the bacterial cell surface to prevent phage attachment. Bacteria can alter or mask receptor molecules, produce extracellular matrix components such as biofilms, or secrete decoy molecules to interfere with phage binding (Dy et al. 2014; Fernández et al. 2018; R. Y. Young 1992). Once a phage penetrates the cell, intracellular mechanisms are activated within the host cell. Systems such as restriction-modification (R-M) and toxin-antitoxin modules protect the bacterial genome by degrading or inactivating foreign DNA, whereas abortive infection (Abi) systems trigger programmed cell death to halt phage propagation (Barrangou and Marraffini 2014; Samson et al. 2013). The CRISPR‒Cas system provides bacteria with adaptive immunity, enabling them to “remember” and neutralize specific phages encountered in the past. This system is a potent bacterial defense mechanism that has been co-opted as a revolutionary tool for genome editing (Esteves and Scharf 2022; Warwick-Dugdale et al. 2019).

In addition to these well-characterized mechanisms, recent research has revealed novel defense systems, including phosphorothioation (P-T), DISARM, and BREX, which similarly modify host DNA and restrict phage replication, alongside prokaryotic Argonautes and viperins that degrade foreign nucleic acids or inhibit viral transcription(Anastassopoulou et al. 2025). Complementing these defenses are NAD+ depletion systems, nucleotide metabolism enzymes, membrane-stabilizing dynamins, and abortive infection modules such as CBASS and retrons, which trigger programmed cell death to limit phage spread. In addition to these well-characterized mechanisms, recent research has revealed novel defense systems, such as bacteriophage exclusion (BREX), defense island systems associated with restriction–modification (DISARM), and phage-inducible chromosomal islands (PICIs), which have expanded our understanding of bacterial strategies against phages(Ge et al. 2025; Rezaei et al. 2025; Vaysset and Bernheim 2025). These discoveries underscore the remarkable ingenuity of bacterial evolution in shaping a complex and dynamic immune network and open new avenues for biotechnological applications, including the development of phage-resistant bacterial strains and novel gene-editing tools (Table 1). These discoveries underscore the remarkable ingenuity of bacterial evolution and open new avenues for biotechnological applications (Abedon 2011; M. R. Clokie and Kropinski 2009; Goldfarb et al. 2015).

**Table 1:** Comprehensive overview of bacterial antiphage defense systems, classified by mechanistic category, genomic prevalence, and mode of action

Defense System	Prevalence in Genomes	Primary Mechanism		Mechanistic Description
*Nucleic Acid–Targeting Systems*				
Restriction–Modification (R-M)	~ 90%	DNA cleavage	Host DNA is methylated; unmethylated phage DNA is recognized and cleaved by restriction endonucleases (Skutel et al. 2023).	
Phosphorothioation (P-T)	> 14.7%	DNA cleavage	Sulfur modification marks host DNA, enabling specific cleavage of unmodified phage DNA. In phosphorothioate (PT)-based Dnd defense systems, the DndABCDE complex catalyzes the incorporation of PT modifications into host double-stranded DNA at specific motifs (5′-GPSAAC-3′/5′-GPSTTC-3′, where PS denotes a phosphate sulfur bond). These sequence-specific PT marks then act as self-recognition signals, enabling the DndFGH complex to identify and degrade invading foreign DNA that lacks such modifications (Jiang et al. 2023; Wang et al. 2019, 2021a, b).	
DISARM	3.5%	DNA cleavage	Multi-protein system combining methylation and nucleases to restrict phage DNA replication (Ofir et al. 2018).	
BREX	10%	DNA cleavage	Host DNA is epigenetically marked; unmodified phage DNA replication is inhibited (Beck et al. 2022; Picton et al. 2021).	
CRISPR–Cas	~ 40%	DNA/RNA cleavage	Acquired spacers guide Cas nucleases for targeted nucleic acid degradation (Bondy-Denomy et al. 2013).	
Prokaryotic Argonaute protein	9%	Nucleic acid degradation / NAD depletion	Prokaryotic Argonaute proteins (pAgos) are conserved defense factors in bacteria and archaea that protect against phage and foreign DNA. Unlike eukaryotic Argonautes, which use RNA guides, many pAgos employ small DNA guides to target and cleave invading nucleic acids. They exist as long forms, which directly mediate antiviral DNA interference, and short forms, which act with partner proteins and can trigger cell death for population-level protection (Bernheim and Sorek 2020).	
Prokaryotic viperins	< 1%	Transcription inhibition	Prokaryotic viperins belong to the radical SAM (S-adenosylmethionine) enzyme family. These enzymes generate reactive radical intermediates that can chemically modify nucleotides or proteins. In the case of viperins, they produce modified nucleotide analogs (such as ddhCTP), which act as chain-terminating inhibitors of viral RNA polymerases. This effectively blocks phage RNA transcription and replication, forming part of the bacterial antiviral defense mechanism. They are frequently located in genomic regions associated with other defense modules, such as toxin–antitoxin systems (e.g., HicA, RelE), suggesting synergistic roles in phage resistance. As part of the broader prokaryotic immune repertoire, including CRISPR–Cas, restriction–modification, and other systems, viperins contribute to multilayered, cooperative defense strategies against phage infection (Bernheim et al. 2021).	
Vibrio integron gene clusters (9 new)	~ 5%	Conditionally expressed from integron arrays	Integron-associated gene cassette clusters encoding diverse proteins, including predicted membrane-associated and intracellular factors. Confers resistance to multiple bacteriophages, likely acting at early stages of infection through interference with phage adsorption or genome entry; precise molecular mechanisms remain to be elucidated (Blokesch 2025; Getz et al. 2025).	
TIR-based (Thoeris-like)	~ 6%	Capsid sensing / NAD⁺ depletion	TIR-domain sensor proteins (ThsB homologs) detect phage structural proteins, including major capsid proteins, during infection. Upon activation, the TIR domain catalyzes the synthesis of 1″-3′ glycocyclic ADP-ribose (gcADPR), which functions as a second-messenger molecule to activate the ThsA NADase effector. ThsA-mediated NAD⁺ depletion disrupts host metabolism and halts phage replication, resulting in abortive infection–like antiviral defense (Doron et al. 2018; C. G. Roberts et al. 2025).	
*Toxin–Antitoxin & Abortive Infection Systems*				
Toxin–Antitoxin (TA)	Widely distributed	Translation/DNA replication arrest	Toxin activation blocks essential processes, triggering abortive infection (Guegler and Laub 2021).	
CBASS	> 10%	Cyclic oligonucleotide signaling / cell suicide	Detects phage infection, produces second messengers, induces host suicide to prevent spread (Bernheim and Sorek 2020).	
Retrons	5%	Abortive infection / membrane damage	Produce multicopy ssDNA, activating abortive infection and limiting phage propagation (Millman et al. 2020).	
Dionysus (TerB)	~ 2%		Identified through modularity network analysis as a defense system encoding a TerB homolog; experimental validation indicates anti-phage activity, though the precise molecular mechanism and target phage families remain to be characterized (van den Berg et al. 2025).	
Pycsar	Rare	NAD depletion / membrane degradation	Disrupts NAD+ pools or membrane integrity; mechanism under study (Tal et al. 2021).	
*NAD+ Depletion Systems*				
Thoeris	4%	NAD+ depletion	Detects phage proteins and depletes NAD+, disrupting metabolism (Doron et al. 2018).	
DSR	Rare	NAD+ depletion	Induces NAD+ depletion following infection; details remain unclear (Garb et al. 2022).	
Mobilome-associated (S. aureus)		SaPI2 mobilome carries stl2, encoding a transcriptional repressor that oligomerizes into multimers	Stl2 binds and inhibits phage-encoded homologous recombinases (e.g., Sak, Sak4) by forming oligomeric complexes that decorate HR structures, preventing recombination activity essential for phage DNA replication and thereby conferring robust antiviral immunity(Debiasi-Anders et al. 2025).	
SEFIR	1.2%	NAD+ depletion	Encodes NAD+-consuming enzymes, impairing phage replication (Millman et al. 2022).	
*Nucleotide Metabolism Systems*				
Nucleotide-manipulating enzymes	> 2.5%	Nucleotide depletion	Degrade or modify nucleotide pools to block phage DNA synthesis (Tal et al. 2022).	
*Membrane-Targeting Systems*				
Dynamins	1.3%	Membrane remodeling	Stabilize membrane integrity, delaying phage-induced lysis (Millman et al. 2022).	
*Emerging / Poorly Characterized Systems*				
Gabija	8.5%	Unknown (possible DNA modification)	Recently characterized, associated with DNA repair/modification enzymes (Doron et al. 2018).	
Lamassu family	11.7%	Unknown	Found in defense gene clusters; mechanism under study (Millman et al. 2022).	
SOFIC	13.8%	Unknown	Highly prevalent, function not yet resolved (Millman et al. 2022).	
Zorya II/III, Druantia III	~ 4%	Synergistic multi-gene	Enhanced anti-phage activity when both systems are present, indicating synergistic defense against phage infection; individual mechanisms not fully elucidated(Wu et al. 2024).	
Hachiman, Shedu, Septu, Kiwa,, Wadjet,, ISG15-like, Mokosh, Azaca, Olokun, Menshen, Dazbog, Uzume, Nhi, Bunzi, Shango, Tiamat, Dodola, Borvo, Aditi	0.2–8.6%	Unknown	Diverse systems found in defense islands; mechanisms range from abortive infection to genome defense, largely uncharacterized (Doron et al. 2018; Millman et al. 2022).	

Well-characterized systems (e.g., R–M, CRISPR–Cas, DISARM, BREX) primarily target nucleic acids, whereas others function via toxin–antitoxin modules, NAD⁺ depletion, nucleotide metabolism, or membrane stabilization. Numerous recently described systems remain poorly characterized, underscoring the diversity and complexity of prokaryotic antiviral immunity

We explore each category of bacterial defense mechanisms in detail, examining their molecular foundations, evolutionary significance, and potential implications for medicine and biotechnology.

## Surface-based defenses

Phage infection is initiated through adsorption to specific bacterial surface receptors. These receptors are essential bacterial surface structures that play crucial roles in physiological processes, including nutrient uptake, motility, and communication. However, their accessibility makes them ideal targets for phages, which exploit these molecules to attach, inject their genetic material, and initiate their replication cycle.

Phage receptors are highly diverse and include proteins, polysaccharides, glycoproteins, and phospholipids. The specificity of these receptors often dictates the host range of a phage, as the phage must evolve receptor-binding proteins (RBPs) that precisely match the receptor’s structure (Bebeacua et al. 2013; Henderson et al. 1996). To counteract this, bacteria have evolved various innate defense mechanisms that modify or mask these surface structures, effectively blocking phage attachment and subsequent infections. These surface modifications form a critical first line of defense and highlight the adaptability of bacteria to evolutionary pressure (Hasan and Ahn 2022).Depending on the bacteriophage and host, the recognition of receptors based on their location and nature varies greatly(Bertozzi Silva et al. 2016). For instance, bacteriophages bind to receptors located in lipopolysaccharide (LPS) walls, which are exclusively observed in gram-negative bacteria (Marti et al. 2013); teichoic acid in Gram-positive bacteria; bacterial capsules or slime layers (Fehmel et al. 1975); and appendages such as pili, fimbriae (Guerrero-Ferreira et al. 2011) and flagella (Bertozzi Silva et al. 2016).

Phage adsorption frequently proceeds through multireceptor mechanisms involving sequential reversible and irreversible interactions(Carroll-Portillo and Lin 2019). In this process, phages commonly employ distinct proteins for reversible and irreversible binding. Notably, phage T5, which infects *Escherichia coli*, initially binds reversibly to the polymannose moiety of the host’s lipopolysaccharide (LPS) via its L-shaped fibers. Subsequently, irreversible adsorption occurs by interaction of the phage tail protein pb5 with the outer membrane receptor protein FhuA (Heller and Braun 1982). Similarly, phage T4 exhibits a two-step binding mechanism in which its long tail fibers mediate reversible adsorption onto LPS, followed by irreversible binding of short tail fibers to the heptose moiety of LPS (Riede 1987). Comparable adsorption principles have been observed in Gram-positive bacteria. For example, phage SPP1, which targets *Bacillus subtilis*, initially binds to the host’s glucosylated wall teichoic acid (WTA) in a reversible manner. Irreversible adsorption is then achieved through the interaction between the phage gp21 protein and YueB membrane protein receptor (Baptista et al. 2008). This stepwise mechanism, starting with reversible binding to more exposed and accessible cell surface moieties, enhances the stability of the phage‒host interaction and increases the likelihood of successful targeting of receptors critical for irreversible binding (Chatterjee and Rothenberg 2012; Garen and Puck 1951).By adopting such strategies, phages maximize their ability to locate and securely attach to host bacteria, leveraging exposed surface molecules for initial engagement before progressing to specific, high-affinity interactions. This diversity in the receptors and structures involved is a test of the multiplicity of mechanisms developed by phages and their hosts to overcome the evolutionary strategies adopted by their counterparts.

### Receptor modifications

#### Lipopolysaccharide (LPS)

Lipopolysaccharide (LPS) is an essential component of the outer membrane of gram-negative bacteria, and it plays a crucial role in bacterial interactions with the environment, including interactions with bacteriophages. The LPS structure, which is composed of lipid A, a core oligosaccharide, and an O-antigen polysaccharide, often serves as the primary receptor for phage attachment (Caroff and Novikov 2019; Paracini et al. 2022). For example, *Pseudomonas aeruginosa* expresses three types of O-antigens, which include the common polysaccharide antigen (CPA; homopolymer O-antigen), O-antigen specific (OSA; heteropolymer O-antigen), and uncapped antigen (no or one O-antigen sugar). Therefore, resistance mutations for LPS-exploiting phages vary according to the LPS component or type of phage that specifically binds (Vaitekenas et al. 2021). Because phages are highly specific for host recognition, modifications in LPS can serve as an effective defense mechanism against phage infection by preventing or reducing phage adsorption on the bacterial surface (Winstanley et al. 2016).

##### Structural modifications of the O-antigen

One of the most common and adaptive bacterial strategies to avoid phage infection is structural modification of the O-antigen, the most variable region of the LPS (Paracini et al. 2022). This region is often the target of phages, as it is exposed on the bacterial surface and can serve as the binding site for the phage’s receptor-binding proteins (RBPs). Bacteria can alter the O-antigen structure to prevent phage adsorption through various enzymatic modifications. For example, *Salmonella enterica* uses glycosyltransferases such as WbbL and Wzy to incorporate unique sugars, such as N-acetylgalactosamine or rhamnose, into the O-antigen, disrupting the ability of phages to recognize and bind to these surface structures (Gasperini et al. 2024; Reeves et al. 2013). Moreover, phase variation, a genetic mechanism in which bacteria can switch between different O-antigen structures, allows bacteria to dynamically alter their surface characteristics. This variability ensures that some bacteria in the population may be susceptible to phages, whereas others can escape infection because of the altered O-antigen structure(Winstanley et al. 2016).

##### Chemical modifications of the LPS core region

In addition to causing structural changes in the O-antigen, bacteria can chemically modify other parts of the LPS molecule, such as the core oligosaccharides and lipid A. These modifications can alter the overall charge, hydrophobicity, or structural integrity of LPS, reducing the ability of phages to bind to the bacterial surface. For example, *Pseudomonas aeruginosa* adds glycine residues to the lipid A portion of LPS, which reduces the binding affinity of phages that target this receptor (Moskowitz et al. 2004). Another important modification is the phosphorylation of lipid A, which can be mediated by enzymes such as LpxT. Phosphorylation of lipid A protects *Escherichia coli* from phage infections by altering the surface properties of the bacterium and hindering phage binding (Bishop 2008; Sharma et al. 2016). Moreover, the acetylation of lipid A, which is catalyzed by enzymes such as PagP, is another common modification that can protect bacteria from phages that rely on LPS for infection (Dixon and Darveau 2005; Giordano et al. 2020). These modifications help bacteria evade phages by reducing the accessibility of their LPS structures to the phage’s receptor-binding proteins.

#### Teichoic acid

Teichoic acids are anionic glycopolymers that are integral to the cell walls of Gram-positive bacteria and play essential roles in maintaining cell wall integrity, regulating ion homeostasis, and mediating interactions with external molecules (Brown et al. 2013). While crucial for bacterial physiology, these structures often serve as receptors for bacteriophages during the infection. Consequently, bacteria have evolved various defense mechanisms to modify their teichoic acids and evade phage adsorption. These modifications highlight the adaptability of bacterial systems under selective pressure from phages, underscoring the ongoing arms race between hosts and pathogens.

Structurally, there are two main types of teichoic acids: wall teichoic acids (WTAs), which are covalently attached to the peptidoglycan layer, and lipoteichoic acids (LTAs), which are anchored to the cytoplasmic membrane(Brown et al. 2013). These structures often present species-specific and strain-specific glycosylation patterns, which bacteriophages exploit for binding. For example, WTAs in *Staphylococcus aureus* are composed of ribitol phosphate repeating units substituted with D-alanine or sugar residues, which phages recognize during the adsorption process (Brown et al. 2013; van Dalen et al. 2020). In the case of *Bacillus subtilis*, phage SPP1 binds reversibly to glucosylated WTAs, subsequently transitioning to irreversible attachment through interactions with the membrane protein YueB (Vinga et al. 2012).

Phages often use a two-step binding mechanism to ensure successful adsorption of the host. The reversible binding stage allows them to anchor onto surface-exposed moieties such as WTAs, providing stability while searching for specific receptors. This strategy increases the likelihood of efficient and irreversible attachment, enabling subsequent DNA injection into the host cell. However, bacteria can disrupt this critical step through targeted modifications to their teichoic acids. Teichoic acid modifications represent a sophisticated and highly effective bacterial defense mechanism against phages. By altering their WTAs through glycosylation, D-alanylation, phase variation, and enzymatic degradation, Gram-positive bacteria can prevent phage adsorption and infection. These adaptations illustrate the dynamic interplay between bacterial survival strategies and phage evolution (Brown et al. 2013).

#### Modifications to teichoic acids for phage defense

##### Glycosylation of teichoic acids

This is a common strategy of the bacterial defense mechanism to evade bacteriophages by masking phage-binding sites. Here, glycosylation of WTAs involves the attachment of various sugar residues, such as glucose or N-acetylglucosamine, to teichoic acids, effectively masking phage-binding sites. This mechanism is exemplified by glycosylated-mediated defense mechanism in *Staphylococcus aureus*. This process is mediated by enzymes such as TarM and TarS, which add N-acetylglucosamine to WTAs, thereby reducing the binding efficiency of specific phages (Leprince and Mahillon 2023; Stone et al. 2019). In some cases, glycosylation patterns are dynamic, allowing bacteria to evade a broader range of phages. This flexibility highlights the evolutionary adaptability of bacteria to the persistent predation of phages. For example, *Listeria monocytogenes* modifies its WTAs by altering sugar moieties, which significantly reduces phage adsorption and improves bacterial survival (Sumrall et al. 2020).

##### D-alanylation of teichoic acids

In addition to glycosylation, the incorporation of D-alanine into teichoic acids, a process regulated by the Dlt operon, plays an essential role in the defense mechanism. D-alanylation decreases the negative charge of WTAs and LTAs, thereby reducing the electrostatic interactions with positively charged phage proteins. This modification not only hinders phage adsorption but also enhances bacterial resistance to antimicrobial peptides, offering a dual protective advantage (Leprince and Mahillon 2023). Studies have shown that *Staphylococcus aureus* strains lacking D-alanine modifications are more susceptible to phage-mediated lysis (Munita and Arias 2016).

##### Enzymatic degradation of teichoic acids

Bacteria can also enzymatically degrade or remodel WTAsto disrupt phage binding. This strategy involves the removal of specific residues that serve as phage-binding sites, rendering the bacteria less susceptible to infection. For example, *Listeria monocytogenes* produces enzymes that cleave key components of WTAs, reducing the effectiveness of phage adsorption (Carvalho et al. 2014; Denes et al. 2015).

In addition to the above-discussed mechanism, some bacteria exhibit phase variation, a reversible mechanism that alters the glycosylation or composition of teichoic acids. This strategy allows bacteria to switch between different structural states, some of which may render them resistant to specific bacteriophages. In *Bacillus subtilis*, phase variation in WTA glucosylation affects phage SPP1 adsorption, enabling bacteria to escape infection. Specifically, phage SPP1, which infects *Bacillus subtilis*, recognizes glucosylated WTAs during the initial stages of infection. Mutations in genes responsible for WTA glucosylation, such as *tagO and gtaB*, significantly reduce phage binding and infectivity (Vinga et al. 2012). This modification alters the bacterial cell surface, preventing the adsorption of certain phages by obstructing the receptor binding sites. This modification also influences the overall fitness of bacteria, as it requires balancing phage resistance with metabolic costs (Xia et al. 2010). Phase variation provides a survival advantage by diversifying the bacterial population and increasing the likelihood of survival under phage pressure.

### Extracellular structures: capsules and biofilms

Bacterial cells often rely on extracellular structures, such as capsules and biofilms, as physical and biochemical barriers against external threats, including bacteriophage infections. These surface-associated structures provide robust defenses, obstructing phage adsorption and impeding their ability to establish infection. This multifaceted resistance is a critical survival strategy for bacteria in diverse environments and exemplifies the co-evolutionary arms race between phages and their bacterial hosts.

Capsules are dense, often polysaccharide-rich layers surrounding bacterial cells, functioning as the first line of defense against phages. They form a physical barrier and block access to specific receptors on the bacterial surface, preventing phage adsorption and subsequent infection. Capsular polysaccharides are highly variable among bacterial species, contributing to strain-specific resistance (Shabbir et al. 2016; Sørensen et al. 2021). For example, the capsule of *Escherichia coli K1* effectively prevents the attachment of phages that target the lipopolysaccharides (LPS) underneath the capsule (Scholl et al. 2005). However, this defense is counterbalanced by the emergence of K1-specific phages (e.g., K1F and K1-5) that encode capsule-degrading endosialidases, illustrating a highly specialized co-evolutionary interaction between the two.

A comparable yet more complex capsule-mediated defense strategy is observed in *Klebsiella pneumoniae*, a pathogen characterized by exceptional capsular diversity encompassing more than 80 distinct serotypes. In *K. pneumoniae*, the thick polysaccharide capsule impedes phage adsorption by shielding conserved outer membrane structures and enhances resistance to immune clearance, antimicrobial peptides, and environmental stress. This extensive capsular heterogeneity significantly restricts the ability of phages to evolve into broadly effective receptor-binding proteins, thereby limiting the phage host range and infection efficiency (Y.-J. Pan et al. 2019a, b). Although certain *K. pneumoniae*-specific phages encode depolymerases capable of degrading defined capsule types, resistance frequently emerges through capsule modification, downregulation, or complete loss of capsule synthesis. While such adaptations reduce phage susceptibility, they often incur fitness and virulence trade-offs, underscoring the evolutionary balance between capsule-mediated defense and pathogenicity.

Another extracellular structural defense mechanism involves the biofilm formation. Biofilms are complex, multicellular communities of bacteria encased in an extracellular polymeric substance (EPS) matrix, which consists of polysaccharides, proteins, lipids, and extracellular DNA (eDNA). Biofilms are a hallmark of bacterial survival in hostile environments, providing resistance to antibiotics, host immune responses and phage predation. The EPS matrix acts as a physical and chemical shield, trapping phages and preventing their penetration into deeper layers of the biofilm(Karygianni et al. 2020; Wang et al. 2023a, b, c).

The structural complexity of biofilms presents multiple barriers to phages. In *Pseudomonas aeruginosa* biofilms, the EPS component alginate plays a critical role in restricting phage diffusion and adsorption (Hanlon et al. 2001). Similarly, *Staphylococcus epidermidis* biofilms produce poly-N-acetylglucosamine, which enhances resistance to phages by limiting access to bacterial receptors (Le et al. 2018).

Biofilm-mediated phage resistance is a multifaceted strategy that protects bacterial communities from phage predation. One primary mechanism is the physical shielding provided by the extracellular polymeric substance (EPS) matrix, which impedes phage diffusion, preventing them from accessing and binding to bacterial receptors (Ferriol-González and Domingo-Calap 2020). This barrier is particularly effective against lytic phages that require direct contact with their host cells for propogation. Studies on *Pseudomonas fluorescens* have demonstrated that biofilms can trap and inactivate phages within the EPS matrix, effectively neutralizing their infectious potential (Dunsing et al. 2019). Additionally, the inherent phenotypic and genetic heterogeneity within biofilm communities further enhances their resistance. Subpopulations within biofilms may downregulate phage receptors or acquire mutations that confer resistance, ensuring that at least a fraction of the bacterial population survives even under intense phage attack (Harper et al. 2014). Another significant defense mechanism involves extracellular DNA (eDNA) within the biofilm matrix, which can bind to and immobilize phages, effectively trapping them and preventing infection. This “phage-trapping” mechanism has been observed in *Pseudomonas aeruginosa* biofilms, where eDNA contributes to phage sequestation, significantly reducing their efficacy (Whitchurch et al. 2002). Together, these mechanisms make biofilms a formidable barrier to phage infection and a key survival strategy for bacterial communities.

### Down-regulation of the receptors

The downregulation of phage receptors on cell surfaces by reducing the expression of these bacterial receptors effectively decreases the likelihood of phage adsorption and subsequent infection. This mechanism plays a crucial role in bacterial survival and is often regulated by quorum sensing and other metabolic adaptations.One of the primary ways in which bacteria regulate the expression of phage receptors is through quorum sensing, a system that adjusts gene expression based onbased on cell population density. For example, in *Vibrio anguillarum*, quorum sensing downregulates the expression of the OmpK receptor at high cell densities, thereby reducing the susceptibility of the bacterium to phage KVP40 (Tan et al. 2015). A similar mechanism has been observed in *Escherichia coli*, where quorum-sensing signals lead to a reduction in the number of λ phage receptors on the bacterial surface (Ding et al. 2021; Wang et al. 2022). This significantly decreases the rate of adsorption. By modulating receptor expression in response to population density, bacteria can limit phage infections when they are most vulnerable while optimizing receptor availability under conditions that favor growth and resource acquisition.

In addition to quorum sensing, bacteria employ non-mutational defense mechanisms to evade phage infection. Unlike genetic mutations that permanently alter receptor structure, non-mutational strategies allow bacteria to reversibly modify their surface receptors in response to environmental cues. For example, *Vibrio alginolyticus* downregulates specific receptors and transporters in its membrane to avoid infection by lytic bacteriophages (Skliros et al. 2023). This form of defense is part of a broader metabolic adaptation that includes changes in quorum-sensing regulatory proteins and other cellular processes. By selectively suppressing the expression of receptors required for phage adsorption, bacteria can maintain their overall fitness while mitigating the risk of phage attacks.However the downregulation of phage receptors has broader implications for bacterial physiology and virulence. In some cases, the same receptors that phages exploit for infection also play essential roles in nutrient uptake, biofilm formation, or interactions with host organisms. In *Vibrio anguillarum*, for example, phage-resistant variants that downregulate the OmpK receptor exhibit reduced virulence and altered biofilm formation. This suggests that receptor downregulation, although beneficial for phage resistance, involves trade-offs that may affect bacterial survival in other contexts. Similarly, in *Vibrio alginolyticus*, changes in receptor expression have been linked to modifications in quorum-sensing pathways, indicating that phage resistance strategies can influence broader regulatory networks within bacterial populations (Høyland-Kroghsbo et al. 2013).

The trade-offs associated with receptor downregulation highlight the complex interplay between bacterial defense mechanisms and their ecological fitness. While reducing receptor expression helps bacteria evade phage infection, it can also impose metabolic costs and limit their ability to exploit certain environmental resources. These trade-offs underscore the dynamic nature of bacterial evolution, where survival strategies must balance the immediate threat of phage predation with the long-term need for growth and adaptation.While surface-associated defenses serve as the primary barrier limiting phage attachment and entry, intracellular defense mechanisms are activated as a secondary protective layer upon successful phage genome delivery into the bacterial cytoplasm.

## Intracellular defense mechanisms

While surface and biofilm-mediated defenses act as the initial barriers against phages, bacteria also possess intracellular mechanisms to neutralize phages that successfully bypass these external defenses. These systems target the phage lifecycle at the DNA or RNA level, disrupting its replication and, in some cases, sacrificing the infected bacterial cell to protect the broader population. Three primary categories of intracellular defenses include Restriction-Modification (R-M) systems, abortive infection (Abi) systems, and nucleotide-based immunity systems such as Bacteriophage Exclusion (BREX) and Defense Island System Associated with Restriction-Modification (DISARM) (Picton et al. 2021). These systems exemplify the layered and interconnected strategies bacteria use to ensure survival.

### Restriction-modification (R-M)

**Fig. 4:**
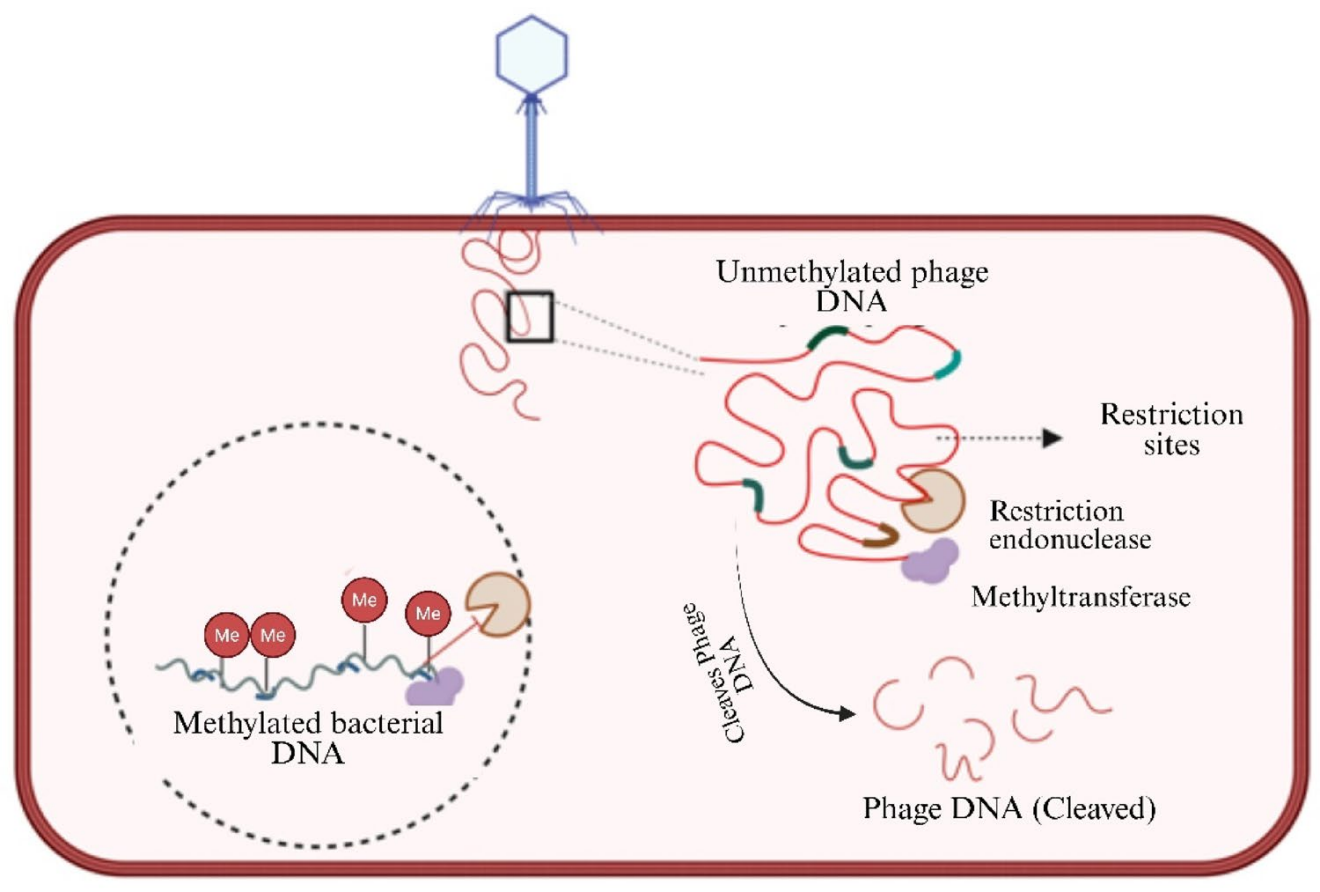
R-M bacterial defense mechanism. Bacterial restriction-modification system defending against bacteriophage infection. The image illustrates how bacterial DNA is protected through methylation, while unmethylated phage DNA is recognized and cleaved by restriction endonucleases at specific restriction sites, preventing the phage from replicating Created in BioRender. Aranjani, J. (2026) https://BioRender.com/tc69wi2

Restriction-modification (R-M) systems are the cornerstone of bacterial intracellular defense, offering a highly specific mechanism to recognize and degrade foreign DNA, including phage genomes. These systems consist of two main components: restriction endonucleases that cleave DNA at specific recognition sequences and methyltransferases that modify the bacterial genome by adding methyl groups to specific bases, thus distinguishing self-DNA from non-self-DNA (Fig. 4).

R-M systems are classified into four types based onbased on their structure, mode of action, and recognition sequence complexity. Type II R‒M systems, such as EcoRI in *Escherichia coli*, have been extensively studied for their precise cleavage activity and wide application in molecular biology (R. J. Roberts et al. 2003). Similiar to EcoKI system, type I systems combine restriction and modification activities within a complex enzyme and require ATP for their function, whereas type III systems, such as EcoP15I, require two recognition sites to trigger cleavage (Murray 2000). Type IV systems add another layer of protection by targeting modified DNA, such as methylated or hydroxymethylated DNA, which is characteristic of certain phages (Loenen et al. 2014). For example, the PvuRts1I enzyme specifically recognizes glucosylated hydroxymethyl cytosine, a modification found in phage T4 DNA.These systems provide a robust initial response to invading phages, effectively degrading the viral genome and halting infection. However, their specificity also imposes limitations, as mutations in phage DNA sequences or methylation patterns can render R-M systems ineffective, driving the coevolution of bacterial defenses and phage counter-defenses.

### Abortive infection (Abi) systems

Abortive infection (Abi) systems operate as a last-resort defense mechanism, sacrificing infected bacterial cells to prevent the spread of phages within a population. These altruistic systems disrupt critical cellular processes following phage infection, often leading to host cell death but ensuring that progeny phages are not released(Aframian and Eldar 2023; Lopatina et al. 2020). This strategy protects the surrounding bacterial population by halting viral spread. Abi systems achieve this by distrupting essential cellular processes, including translation, transcription, and replication, or by inducing membrane leakage(Fineran et al. 2009). However, the precise mechanisms by which bacteria recognize phage infection to trigger the Abi response remain largely unknown.

#### Classical Abi mechanisms

A classic example of an Abi system is the RexAB system in *Escherichia coli*, which monitors phage DNA replication. When aberrant DNA structures are detected, the system triggers a lethal response, halting cell division and killing the host to contain the infection. Another example is the ToxIN system in *Pseudomonas fluorescens*, which comprises a toxin‒antitoxin pair. Upon phage infection, the antitoxin is degraded, releasing the toxin to inhibit translation and induce cell death (Fineran et al. 2009). Similarly, the AbiZ system in *Lactococcus lactis* involves a protein that blocks phage DNA replication, effectively neutralizing the infection (Chopin et al. 2005). The diversity of Abi systems highlights their adaptability to different phage threats. Although these mechanisms lead to the loss of individual bacterial cells, they provide population-level immunity, ensuring the survival of bacterial colonies.

Abi systems are widespread among bacteria, although they have been predominantly studied in *E. coli* and *Lactococcus. lactis*, a Gram-positive bacterium commonly used in dairy production. In *E. coli*, the Lit and PrrC systems are well characterized. The Lit protease of *E. coli* K12 is activated by the Gol peptide of the T4 major capsid protein during the late stage of infection, leading to cleavage of the elongation factor EF-Tu and resulting in translation arrest. Similarly, the PrrC system in *E. coli* CT196 cleaves tRNA^Lys, depleting the tRNA pool and inhibiting translation, thereby preventing phage replication (Kaufmann 2000; Rostøl and Marraffini 2019).In *L. lactis*, over 20 different Abi systems have been identified, with varying mechanisms of action. For example, the AbiK system significantly reduces phage infectivity by interfering with phage recombination through its polymerase activity and synthesizing long DNA molecules with random sequences. Phages that evade AbiK contain mutations in phage-encoded recombinases, suggesting that AbiK disrupts recombination and hinders phage maturation. Another system, AbiZ, reduces phage burst size by interacting with the phage-encoded holin protein, leading to premature lysis and the release of non-infectious viral particles (Du 2023).

Recent research has revealed kinase-mediated Abi mechanisms in *Staphylococcus epidermidis*, a common skin bacterium. The serine/threonine kinase Stk2 phosphorylates multiple cellular targets upon phage infection, disrupting fundamental processes such as transcription, translation, and metabolism. Phages that evade Stk2-mediated defense often carry mutations in the pacK gene, which triggers Stk2 autophosphorylation, initiating the defense response (Teklemariam et al. 2023).

Abi systems share functional similarities with TA systems, which are another bacterial defense mechanism. Several TA systems, such as MazF/MazE in *E. coli*, inhibit phage infections by degrading RNA transcripts during phage invasion. Some phages have evolved countermeasures, such as the ADP-ribosyltransferase Alt in phage T4, which inhibits MazF toxin. The ability of phages to neutralize TA systems highlights the ongoing evolutionary arms race between bacteria and their viral predators (Fineran et al. 2009).

While Abi systems predominantly lead to bacterial cell death, TA systems may allow reversible dormancy, enabling some cells to recover once the threat subsides. The outcome of these defense strategies depends on various factors, including the specific toxin mechanism, the duration of toxin activity, and the phage’s life cycle. In addition to protein-mediated mechanisms, many Abi systems employ metabolic and molecular interference as an integral strategy, wherein the disruption of essential metabolites and signaling pathways, such as NAD⁺ depletion and cyclic oligonucleotide signaling, enhances the abortive response, ensuring that phage replication is effectively halted within the infected cells.

#### Metabolic and molecular interference in Abi systems

**Fig. 5:**
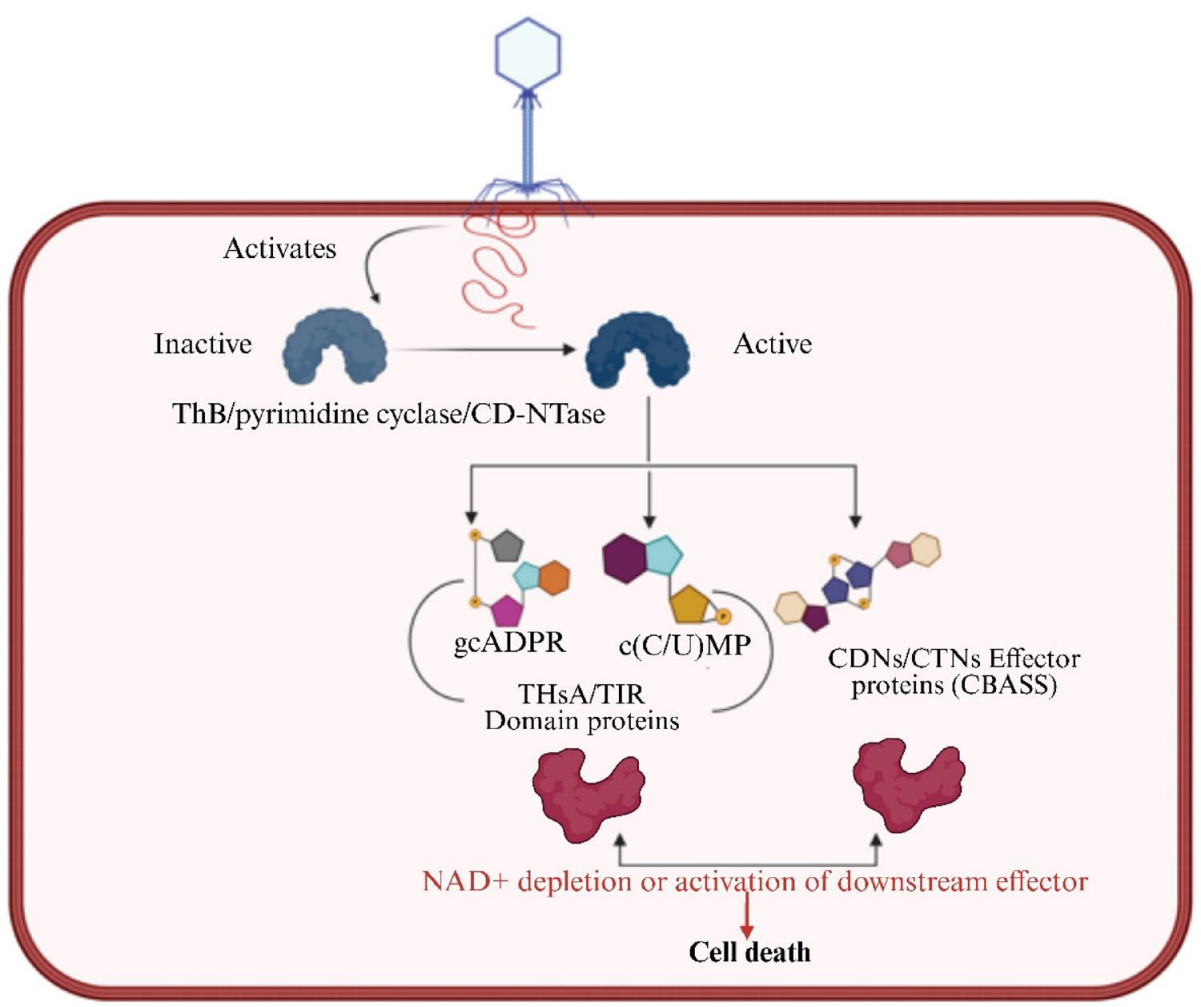
Metabolic and molecular interference mechanism of bacterial defense. Created in BioRender. Aranjani, J. (2026) https://BioRender.com/tc69wi2

Bacteria utilize various NAD+ depletion-based defense mechanisms to counteract bacteriophage infections, preventing their replication and spread. By depleting NAD+, bacteria create a hostile environment that prevents phages from successfully propagating within the cells. Various bacterial immune systems leverage this strategy to eliminate phage-infected cells and protect the bacterial population (M. Wang et al. 2023a, b, c).One key mechanism involved in NAD+ depletion is the action of defense-associated sirtuins (DSRs). These proteins act as NADases that degrade NAD+ upon phage infection, leading to abortive infection and preventing the phage from replicating. In *Bacillus subtilis*, the DSR2 protein recognizes phage tail tube proteins, which activates its NADase activity, rapidly depleting NAD + and stopping phage propagation (Fig. 5). Some phages have evolved counter-defense mechanisms by encoding anti-DSR2 proteins, such as DSR anti-defense 1 (DSAD1), to inhibit the enzymatic activity of DSR2 and evade bacterial immunity(Yin et al. 2024).

Another bacterial defense system that utilizes NAD+ depletion is the Thoeris system. This system involves the synthesis of cyclic ADP-ribose (gcADPR) by ThsB, which activates the ThsA effector. Once activated, ThsA depletes cellular NAD+, preventing phages from utilizing this crucial metabolite for their replication. Similarly, the SIR2-HerA complex functions as a two-protein system that converts the SIR2 protein from a nuclease to an NAD+ hydrolase upon phage infection. This transformation leads to bacterial cell death, ensuring that infected cells do not serve as replication hubs for invading phages (Shi et al. 2024).

The cyclic oligonucleotide-based antiphage signaling system (CBASS) is another highly effective bacterial defense strategy. CBASS functions as an innate immune system that induces cell suicide in response to phage infection, thereby halting the spread of viral particles. This system relies on cGAS/DncV-like nucleotidyltransferases (CD-NTases) to synthesize cyclic oligonucleotides (cOs), which serve as secondary messengers. These cOs activate CD-NTase-associated proteins (Caps) that execute cell death through DNA cleavage, membrane damage, and NAD+ depletion. The collective effect of these mechanisms is the inhibition of phage replication. Notably, these cyclic oligonucleotide signaling molecules are also employed by other bacterial defense systems, including certain CRISPR–Cas variants, underscoring the interconnectedness and complexity of bacterial “pan-immune” networks. However, in response to CBASS, some phages have evolved immune evasion proteins that interfere with Cap recognition by cOs, allowing them to bypass bacterial defense mechanisms and continue their replication cycle (Y. Wang et al. 2023a, b, c).

In addition to protein-based Abi systems, bacteria, particularly *Actinomycetes*, have evolved chemically mediated antiphage defenses in the form of naturally produced secondary metabolites that interfere with multiple stages of the phage infection cycle. Small molecules hinder phage propagation by targeting several stages of the phage infection cycle, such as neutralizing free phage particles and blocking genome entry, transcription, replication, maturation, and lysis. One of the earliest and most effective defense strategies is the inactivation of free phage particles. By preventing the propagation of phages, bacteria can halt infection in its early stages. Anthracyclines, which intercalate into DNA and are produced by *Streptomyces* species, inactivate the *E. coli* phages phiX174 and lambda by disrupting their DNA(Morita et al. 1979).

In the early stages of phage infection, the genome entry process represents another vulnerable target. Actinomycin D, a cyclic peptide from *Streptomyces*, impedes the uptake and retention of phage DNA in *Bacillus subtilis*, resulting in more labeled DNA remaining in the extracellular medium, thus preventing successful infection (Pritikin and Reiter 1969). After genome entry, the replication phase of the phage life cycle can also be inhibited by small molecules. Daunorubicin, an anthracycline derived from *Streptomyces*, prevents lysogen formation in the *E. coli* phage lambda (Kronheim et al. 2018). Aminoglycosides, such as kanamycin, hygromycin, and streptomycin, produced by *Streptomyces* and *Micromonospora* block genome circularization and replication in *Mycobacterium tuberculosis* (Jiang et al. 2020). Apramycin, another aminoglycoside, inhibits genome replication in *Streptomyces venezuelae*, with its antiphage effect distinct from its antibacterial properties (Kronheim et al. 2018). Inhibiting phage transcription is another strategy that bacteria employ to protect against viral infection. Modified ribonucleotides produced by prokaryotic viperins (pVips), such as ddhCTP, ddhGTP, and ddhUTP, inhibit viral RNA polymerase, prematurely terminating RNA chains during *E. coli* phage T7 infection (Bernheim et al. 2021). Class I lanthipeptides from *Streptomyces* also inhibit late-stage phage transcription without affecting bacterial viability, further blocking phage replication (Li et al. 2021). During phage maturation, small molecules can disrupt the assembly of viral components, preventing the formation of mature phage particles. Actinomycin D, a *Streptomyces*-derived secondary metabolite, impedes the maturation of T2r and T4 phages in *E. coli* without affecting total RNA, DNA, or protein synthesis. Electron microscopy reveals that defective phages lack packaged genomes and/or tails, indicating that phage maturation has been halted (Korn 1967). The final step of the phage lytic cycle involves host cell lysis for the release of progeny phages. Phagostatin, a substance produced by *Streptomyces* sp. Kuroya F-300, inhibits lysis in *E. coli* infected by phage T3. Infected bacterial cells accumulate intracellular phage particles, but no plaques or viable colonies form, suggesting an abortive infection where phage reproduction is blocked at the final step. This mechanism prevents the release of infectious phages, limiting the spread of the infection. Taken together, these small molecules play a vital role in bacterial defense against phages, highlighting the intricate and multifaceted nature of bacterial resistance mechanisms (Wong and Balskus 2025).

Overall, Abi systems play a crucial role in bacterial defense by preventing phage propagation through programmed cell death. These systems, though primarily found in Gram-positive bacteria such as *L. lactis*, are also present in Gram-negative species such as *E. coli*,* Vibrio cholerae*, and *Shigella dysenteriae*. The discovery of diverse Abi mechanisms, including premature lysis (AbiZ) and interference with phage recombinases (AbiK), underscores the complexity of bacterial antiviral strategies. The interplay among Abi systems, TA systems, and phage-encoded counter-defense mechanisms continues to shape the coevolution of bacteria and their viral adversaries.

### Nucleotide-based immunity systems

Nucleotide-based immunity systems represent a recently discovered class of bacterial defenses that offer broad-spectrum protection against phages. These systems, including BREX and DISARM, are encoded within bacterial genomes and involve complex multiprotein machinery that targets phage DNA during infection.

The BREX system is a widespread bacterial immune defense that operates by modifying specific DNA motifs through methylation, effectively marking the bacterial genome as “self.” This prevents phages from replicating their DNA within host cells. Unlike traditional restriction-modification (R-M) systems, BREX does not degrade or cleave the phage DNA. Instead, it modifies bacterial DNA at non-palindromic TAGGAG motifs through methylation, mediated by BrxX, a DNA methyltransferase. This modification acts as a molecular signature that distinguishes bacterial DNA from foreign genetic material, preventing phage replication(Beck et al. 2022).

BREX typically consists of six core genes, including BrxX, the primary DNA methyltransferase responsible for adenine methylation within specific motifs, and BrxA, a DNA-binding protein that facilitates methylation specificity and DNA recognition. Additional regulatory and structural proteins, BrxB, BrxC, BrxD, and BrxE, contribute to system the function and stability of the system(Goldfarb et al. 2015; Picton et al. 2021). Some phages encode anti-defense proteins to overcome BREX-mediated defenses. For example, T7 phage protein Ocr, mimics DNA and binds to BrxX, inhibiting its methylation activity. This enables the phage to bypass the bacterial immune response and replicate successfully(Li et al. 2024). BREX systems are found in approximately 10% of all sequenced microbial genomes. These genes were classified into six subtypes based onbased on gene composition and organization, illustrating their evolutionary diversity. This system provides robust and non-degradative protection against phage infection, making it an essential component of bacterial defense. For example, BREX systems in *Escherichia coli* and *Bacillus subtilus* have been shown to block a wide range of phages through an undefined but highly effective mechanism (Goldfarb et al. 2015).

The DISARM system represents a sophisticated nucleotide-based defense mechanism that provides broad protection against phages through a combination of DNA methylation and restriction (Athukoralage and White 2022; Dot et al. 2023; Isaev et al. 2021). Unlike BREX, which blocks replication without cleaving DNA, DISARM actively restricts phage DNA for preventing infection. DISARM uses a multiprotein complex to distinguish bacterial DNA from foreign DNA. It modifies bacterial DNA at CCWGG motifs through methylation, preventing the function and stability of the systemby its restriction enzymes (Zhang et al. 2025). When un-methylated phage DNA enters the cell, the system triggers its restriction and degradation, thereby neutralizing the invading genetic material. The DISARM system is composed of five core genes: DrmA and DrmB, which form the core DNA methylation complex responsible for modifying bacterial DNA; a helicase domain that facilitates DNA unwinding during restriction; a phospholipase D (PLD) domain that provides additional enzymatic activity for DNA cleavage; and the DUF1998 domain, a protein of unknown function that is believed to contribute to system stability and phage recognition (Bravo et al. 2022).

DrmAB remains autoinhibited by a trigger loop (TL) within DrmA. Upon binding to unmethylated phage DNA, the TL is dislodged, triggering structural rearrangements that activate the restriction enzyme complex. This results in the targeted degradation of foreign DNA and preventphage replication. Compared with BREX systems, DISARM systems are more widely distributed across bacterial and archaeal species. Although many phages have evolved mechanisms to evade other restriction-modification systems, there is limited evidence of specific phage countermeasures against DISARM, making it a particularly robust immune mechanism (Bravo et al. 2022; Ofir et al. 2018).

Both BREX and DISARM utilize nucleotide-based modifications to defend bacteria against phages but employ distinct strategies to do so. BREX functions by marking bacterial DNA and preventing phage replication through methylation, whereas DISARM takes a more aggressive approach by degrading foreign DNA. BREX relies on BrxX methyltransferase and BrxA for DNA modification, whereas DISARM includes additional enzymatic components such as a helicase domain and a phospholipase D domain (X. Xu and Gu 2024). Phages have evolved countermeasures, such as Ocr proteins, to inhibit these defense systems; however DISARM appears to have fewer known phage evasion strategies. Understanding these systems not only enhances our knowledge of bacterial immunity but also has potential applications in biotechnology, synthetic biology, and phage therapy for the control bacterial infections.

### CRISPR‒cas systems

The clustered regularly interspaced short palindromic repeats (CRISPR) CRISPR-associated proteins (CRISPR‒Cas) system represents an advanced adaptive defense mechanism in bacteria and archaea, offering precise protection against invading genetic elements such as bacteriophages and plasmids. Unlike innate mechanisms, which provide broad and nonspecific defenses, the CRISPR‒Cas system allows bacteria to develop sequence-specific immunity. By incorporating fragments of phage DNA into their genome, bacteria can “remember” past infections and mount targeted responses against recurring threats. This remarkable capability underscores the sophistication and evolutionary success of the CRISPR‒Cas system (Barrangou et al. 2007; Barrangou and Marraffini 2014).

**Fig. 6:**
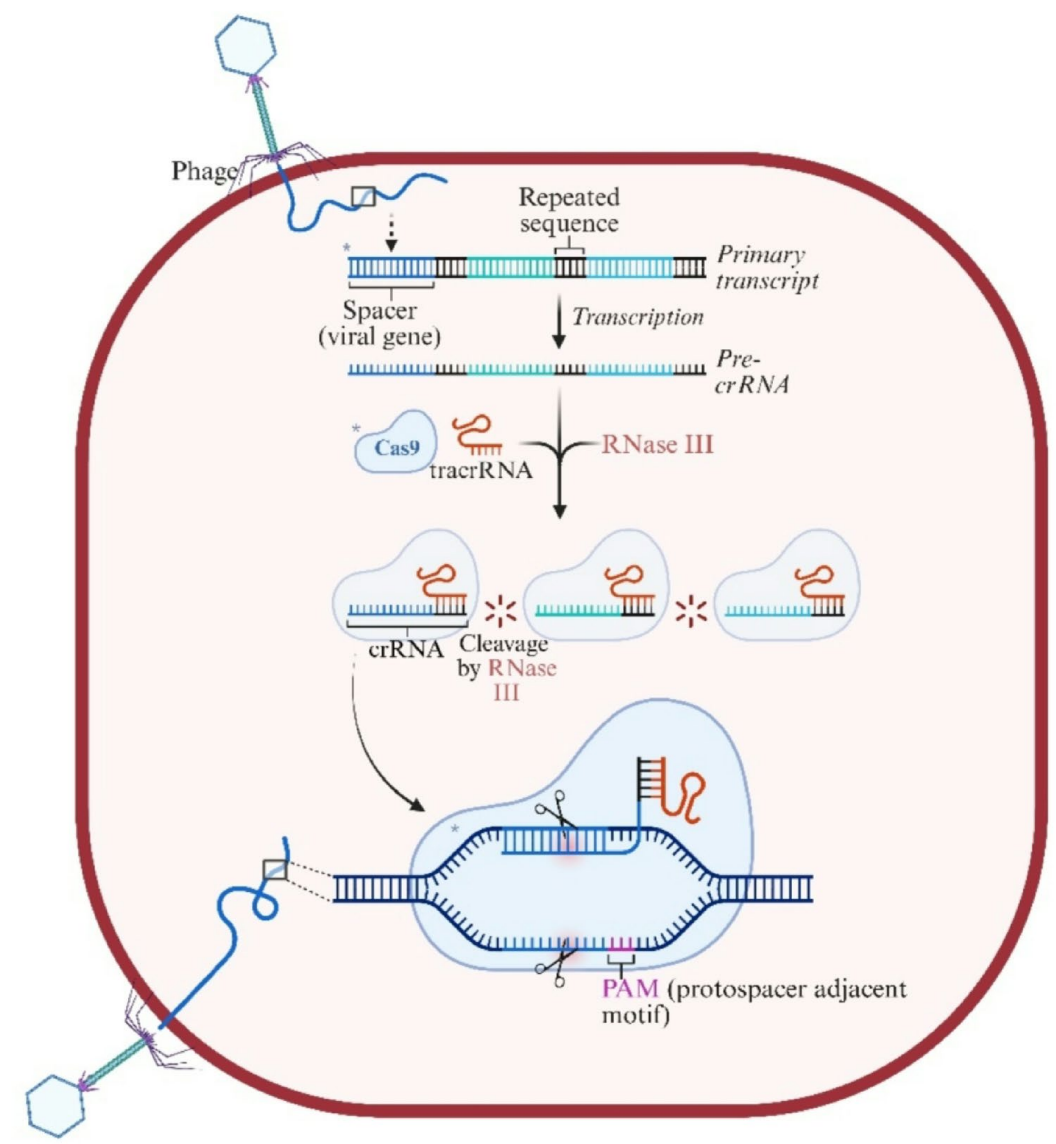
CRISPR‒Cas mechanism of bacterial defense. **a** A bacteriophage attaches to a bacterial cell and injects its DNA. **b** The bacterial CRISPR system incorporates a fragment of phage DNA as a spacer within the CRISPR array. **c** The Cas9 protein complexes with tracrRNA and crRNA to form an RNA-guided surveillance complex. **d** RNase III processes the crRNA-tracrRNA duplex into a functional form. **e** Upon reinfection by the same phage, the CRISPR‒Cas system recognizes foreign DNA and directs Cas9 to cleave it, preventing infection Created in BioRender. Aranjani, J. (2026) https://BioRender.com/tc69wi2

CRISPR‒Cas systems are categorized into two main classes based on their structure and function. Class 1 systems, which include Types I, III, IV, and VII, utilize multiprotein complexes for interference (Makarova et al. 2025). For example, Type I systems use a cascade complex to recognize and cleave foreign DNA. In contrast, Class 2 systems employ a single effector protein such as Cas9 (Type II), Cas12 (Type V), or Cas13 (Type VI) to execute these tasks (Koonin et al. 2017). This simplicity has made Class 2 systems a focal point in genetic engineering and biotechnological applications. The diversity of CRISPR‒Cas systems enables bacteria to tailor their defenses against the vast array of phages they encounter, providing a significant survival advantage to the bacteria.

The CRISPR‒Cas system operates through three interconnected phases: adaptation, crRNA maturation and interference (Ahmed et al. 2024). During the adaptation phase, bacterial cells capture and integrate short DNA fragments (spacers) from invading phages into their CRISPR arrays. This process is mediated by Cas1 and Cas2 proteins and establishes a molecular memory of the infection. In subsequent encounters, the CRISPR array is transcribed into precursor CRISPR RNAs (precrRNAs), which are processed into mature crRNAs. These guide RNAs direct Cas proteins to invading the phage DNA, enabling precise cleavage and neutralization. This process, often termed the interference phase, effectively halts phage infection (Hille et al. 2018). Over time, the CRISPR array expands, maintaining a chronological record of past infections, which forms the basis of adaptive immune memory (Fig. 6).

The CRISPR‒Cas system significantly enhances bacterial immunity by providing specific and durable protection against phages. For example, *Streptococcus pyogenes* employs its Type II CRISPR‒Cas9 system to target and neutralize specific DNA sequences, demonstrating the precision of this mechanism. However, although the system offers many advantages, it is not without limitations. Phages can develop mutations in their target sequences, such as protospacer or protospacer-adjacent motifs (PAMs), allowing them to evade detection and cleavage by CRISPR‒Cas. Additionally, the metabolic cost of maintaining and operating the system may reduce bacterial fitness in environments with low levels of phage pressure. Furthermore, some phages produce anti-CRISPR (Acr) proteins that inhibit CRISPR‒Cas activity, thereby negating their defensive capabilities (Barrangou and Marraffini 2014; Makarova et al. 2020).

Despite these challenges, the CRISPR‒Cas system remains a cornerstone of bacterial defense, demonstrating unparalleled adaptability and specificity (Table 1). The evolutionary arms race between bacteria and phages drives constant innovation in these systems, offering valuable insights into microbial survival strategies. This ongoing conflict has also fuelled advancements in biotechnology, where CRISPR–Cas systems are now harnessed for genome editing, disease modeling, and therapeutic development, further highlighting their importance beyond natural bacterial immunity.

**Table 2:** Bacterial defense mechanisms against phages and corresponding phage counter-defense mechanisms

Sl No	Bacterial Species	Defense Mechanism	Mode of Resistance	Phage Counter-Defense	Reference
1	*Actinobacteria* sp.	Secondary metabolite production	Inhibits phage DNA replication	Adaptation to resist chemical inhibitors	(Nwokolo et al. 2024)
2	*Bacillus subtilis*	DISARM	Degrades unmodified foreign DNA	DNA mimicry	(van Houte et al. 2016)
3	*Bacillus subtilis*	Abortive infection	Bacterial suicide upon infection	Phage evolution to avoid triggering Abi (NADase inhibitor)	(Loyo and Grossman 2024)
4	*Escherichia coli*	CRISPR‒Cas	Sequence-specific cleavage of phage DNA	Anti-CRISPR proteins	(Bondy-Denomy et al. 2013; Makarova et al. 2011)
5	*Escherichia coli*	Superinfection exclusion (Sie)	Prevents entry of secondary phages	Evolution of phages with Sie-resistant pathways	(Bucher and Czyż 2024; Labrie et al. 2010)
6	*Escherichia coli*	MazEF toxin-antitoxin system	Induces dormancy in bacterial populations	Anti-MazE–MazF system	(Engelberg-Kulka et al. 2006; Murtazalieva et al. 2024; Nikolic 2019)
7	*Klebsiella pneumoniae*	Capsule diversity	Prevents phage attachment	Phages targeting conserved capsule components	(Pan et al. 2019a, b; Xu et al. 2024)
8	*Lactococcus lactis*	Phage Inhibitory Protein (PIP)	Blocks phage adsorption on cell surface	Altered receptor-binding proteins	(Mahony et al. 2016)
9	*Lactococcus lactis*	BREX system	Blocks replication of phage DNA through DNA modification		(Goldfarb et al. 2015; Safari et al. 2020)
10	*Mycobacterium smegmatis*	Abi system	Mpr genes, block the entry of phage DNA, over expression leading to abortive infection		(Opperman et al. 2023; Seniya and Jain 2022)
11	*Pseudomonas aeruginosa*	Immune defense mechanism, CRISPR‒Cas	The presence of core defense hotspots (cDHS) which as as anti-phage defense system	Anti-CRISPR‒Cas	(M. C. Johnson et al. 2023; Murtazalieva et al. 2024)
12	*Pseudomonas aeruginosa*	Abortive infection	Toxin-antitoxin system	Phage encodes a gene that closely resembles ToxN antitoxin (‘pseudo-ToxI’) and binds it, inhibiting abortive infection	(Blower et al. 2012; Murtazalieva et al. 2024)
13	*Pseudomonas aeruginosa*	eDNA-mediated resistance	Traps phages using extracellular DNA	Nuclease-producing phages	(Whitchurch et al. 2002)
14	*Pseudomonas aeruginosa*	Biofilm-mediated resistance	Traps phages in EPS matrix	Phage depolymerase	(Juszczuk-Kubiak 2024)
15	*Salmonella enterica*	O-antigen modification	Alters surface receptors to prevent phage adsorption	Phages targeting alternative receptor sites	(Cota et al. 2015)
16	*Staphylococcus aureus*	Abi systems	Bacterial self-destruction to prevent phage replication	The evolution of phages to bypass Abi triggers	(Hasan and Ahn 2022)
17	*Staphylococcus epidermidis*	Poly-N-acetylglucosamine production	Prevents phage receptor access	Phage-encoded EPS depolymerase	(Izano et al. 2008; Nahar et al. 2018)
18	*Streptococcus pyogenes*	Cas9-based DNA cleavage	Targeted DNA cutting of phage genomes	Phage mutations in PAM regions	(Synefiaridou and Veening 2021)
19	*Streptococcus thermophilus*	CRISPR‒Cas	Incorporates phage spacers to target subsequent infections	Phage escape mutations in spacer regions	(Garneau et al. 2010)
20	*Vibrio cholerae*	PLE (Phage Inducible Chromosomal Island)	Hijacks and destroys phage DNA	Phage mutations to evade PLE recognition	(Hays and Seed 2020)

## Bacteriophage counter defense

**Fig. 7:**
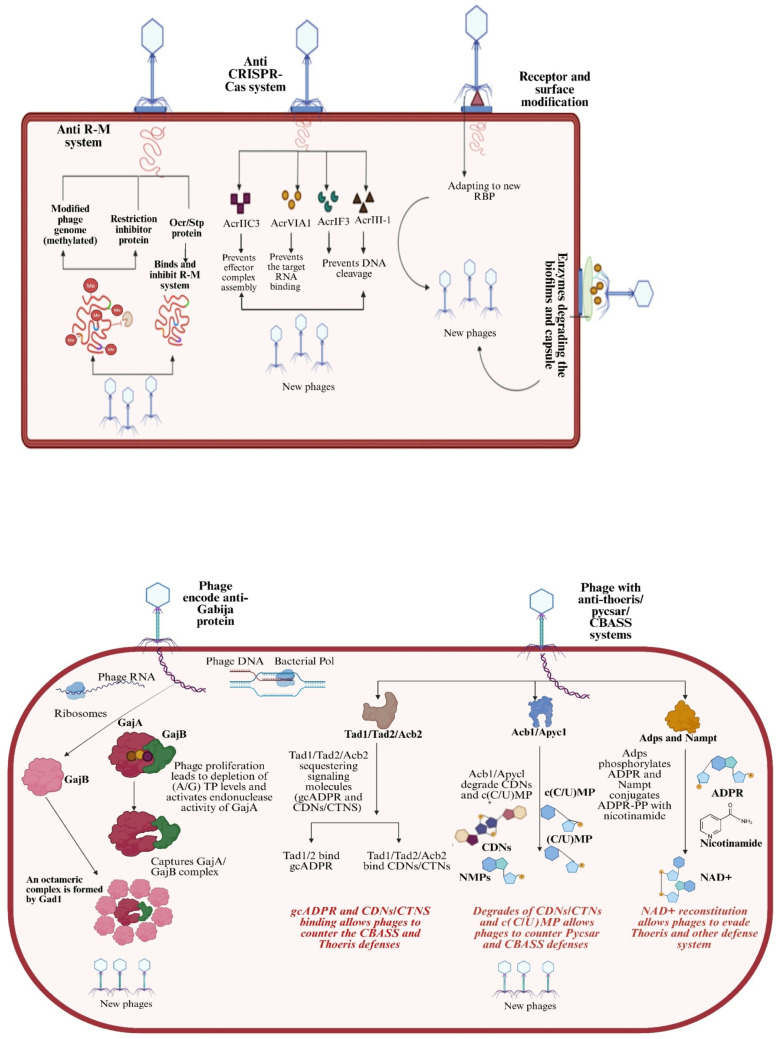
Phage evasion mechanisms against bacterial defense systems. Created in BioRender. Aranjani, J. (2026) https://BioRender.com/tc69wi2

The interaction between bacteria and bacteriophages is a dynamic evolutionary arms race characterized by constant adaptations on both sides (Table 2). While bacteria deploy sophisticated defense mechanisms to prevent phage infections, phages evolve counterstrategies to bypass these defenses and maintain their infective capabilities. These mechanisms demonstrate the adaptability of phages and their critical role in microbial ecosystems (Fig. 7).

### Receptor and surface adaptation

Bacteriophages have evolved multiple strategies to counter bacterial receptor-based defenses. When bacteria modify or mask their receptors to prevent phage attachment, phages respond by adapting to the new receptors via receptor mimicry, breaking down protective extracellular barriers, or evolving to recognize the modified receptors (Dunne et al. 2021; Nobrega et al. 2018).A common approach that phages use to adapt to new receptors by mutating their receptor-binding proteins (RBPs), also known as receptor mimicry, where phages evolve proteins that mimic bacterial receptors, allowing them to bind to and infect bacterial cells despite bacterial resistance mechanisms. Bacteriophages use receptor-binding proteins (RBPs) in their tail fibers or tail spikes to recognize and attach to bacterial surface receptors (de Jonge et al. 2019; Dunne et al. 2019). Tail spikes also contain enzymes that degrade protective polysaccharides, such as capsules or extracellular polysaccharides (EPS), exposing secondary receptors for irreversible adsorption (X. Wang et al. 2021a, b). Podoviridae phages often have depolymerases in their tail spike proteins (TSPs) that breakdown host polysaccharides, allowing DNA injection and infection(Knirel et al. 2020).RBPs function as keys that fit into bacterial receptor locks. A single RBP can recognize multiple receptors, and a single receptor can interact with various RBPs. If a receptor is lost or modified, phage binding is blocked, enabling bacterial resistance to phage infection. However, phages can mutate their RBPs to target new receptorsand adapt to bacterial defenses. The host range of phages can be modified by altering their tail proteins (Philippe et al. 2023; Shuwen and Kefeng 2022).For example, phage λ, which initially binds to the LamB receptor on *E. coli*, can evolve to recognize the OmpF receptor through a series of mutations when LamB expression is reduced (Samson et al. 2013). Similarly, phages can modify their tail fibers or adsorption proteins to recognize alternative receptors when primary receptors are inaccessible. The phage ΦX174 adapts to changes in its host’s lipopolysaccharide (LPS) structure by mutating key proteins involved in attachment (Romeyer Dherbey et al. 2023).

Phages also utilize diversity-generating retroelements (DGRs) to increase receptor adaptability. DGRs consist of reverse transcriptase and two repeat regions: a variable repeat (VR) and a template repeat (TR). Mutations introduced in the VR region create variations in phage tail fiber proteins, allowing phages to recognize different receptors. This mechanism can generate up to 10¹⁴ tail fiber variations, significantly increasing the chances of successful bacterial infections (Benler et al. 2018). Another strategy involves the hydrolysis of extracellular components that shield bacterial receptors. Phages produce enzymes, such as endosialidases, glycosidases, and EPS-degrading enzymes, to break down capsules and exopolysaccharides, exposing hidden receptors for binding(Abd-Allah et al. 2021). Bacterial capsules, composed of polysaccharides, are highly effective barriers against phage adsorption and infection. However, many phages counter this defense by producing capsule-degrading enzymes, such as depolymerases. These enzymes break down the capsular polysaccharides, exposing the underlying receptors and enabling phage attachment. For example, phage K1F uses a specific depolymerase to degrade the capsule of *Escherichia coli* K1, allowing it to bind to its receptor and initiate infection (Scholl et al. 2005). Another example is phage phiKZ, which produces multiple polysaccharide-degrading enzymes that efficiently penetrate bacterial capsules (Cornelissen et al. 2012).

Capsule-degrading enzymes not only facilitate infection but also contribute to the dispersal of bacterial populations, increasing their susceptibility to secondary infections. These findings demonstrate the dual role of these enzymes in overcoming bacterial defense mechanisms and reshaping microbial communities. For example, phage E79 produces an alginate lyase enzyme that breaks down alginate, a key EPS component in *Pseudomonas aeruginosa* biofilms, thereby improving its infectivity and facilitating biofilm disruption (Hanlon et al. 2001).In addition to enzymatic degradation, some phages exhibit enhanced persistence in biofilm environments. This persistence increases the likelihood of phage encounters with susceptible cells, thereby initiating infection. For example, phages targeting *Staphylococcus aureus* biofilms have been shown to persist in the biofilm environment for a long enough period to locate and infect viable cells (Harper et al. 2014). Furthermore, certain phages evolve to preferentially infect biofilm-associated bacteria by recognizing specific biofilm-related receptors or exploiting structural weaknesses in the biofilm.

Through mutational adaptation, enzymatic degradation of bacterial barriers, and receptor-binding protein diversification, phages continuously evolve to overcome bacterial defenses, ensuring their ability to infect host cells despite bacterial resistance strategies. In addition, the genetic engineering of RBPs provides a strategy to expand or change phage specificity, thereby enhancing their potential for antibacterial applications.

### Anti-CRISPR proteins (Acrs)

One of the key counter defenses employed by phages is the production of anti-CRISPR (Acr) proteins. These proteins inhibit bacterial CRISPR‒Cas systems and effectively neutralize their adaptive immunity. Acr proteins function by either blocking phage DNA recognition by the CRISPR complex or preventing DNA cleavage. For example, AcrIIA4 targets the widely studied Cas9 protein of *Streptococcus pyogenes*, directly binds to its nuclease domain, and inhibits its ability to cleave DNA (Pawluk 2016). Another example is AcrIF1, which inhibits the Type I‒F CRISPR‒Cas system in *Pseudomonas aeruginosa* by interfering with the cascade complex responsible for DNA binding (Bondy-Denomy et al. 2013). The diversity of Acr proteins across phages highlights their ability to adapt to different types of CRISPR‒Cas systems, demonstrating the evolutionary pressure exerted by bacterial adaptive immunity. In addition to Acr proteins, some phages encode mini-CRISPR arrays, which produce small CRISPR-derived RNAs that silence host CRISPR–Cas defenses by guiding interference complexes away from phage targets (Camara-Wilpert et al. 2023). Together, these strategies illustrate the diversity of phage anti-CRISPR mechanisms and highlight the evolutionary pressure exerted by bacterial adaptive immunity.

### Anti-restriction modification system

The restriction-modification mechanism is one of the first-line defense mechanisms developed by bacteria. Bacteriophages have developed multiple mechanisms to evade bacterial R–M defenses, including genome modifications, site masking, molecular mimicry, enzyme activation, and cofactor degradation.The key mechanism involves the use of restriction endonucleases (REases) to recognize and cleave specific phage DNA sequences; however phages have developed mechanisms to evade or disable these defenses. One key strategy involvess modifying their genomes with phages to prevent recognition by bacterial REases. Many phages introduce random point mutations in their restriction sites, thereby reducing the likelihood of cleavage. For example, in Bacillus species phages such as SPO1, replacing thymine with hydroxymethyluracil prevents many REases from recognizing phage DNA. Phages also incorporate modified bases such as N6-methyladenine, hydroxymethylcytosine, and glucosylated hydroxymethylcytosine (Drozdz et al. 2012; Krüger and Bickle 1983).

Another common strategy is to mask the restriction sites to protect the phage genome. The coliphage P1, for example, injects DarA and DarB proteins, which bind to its DNA and shield restriction sites from enzymatic cleavage. Some phages also use molecular mimicry to evade restriction systems(Iida et al. 1987). The coliphage T7 produces the Ocr protein, which mimics the DNA phosphate backbone and binds to both the methyltransferase (MTase) and REase domains of the EcoKI enzyme. Ocr has a greater affinity for EcoKI than does phage DNA, leading to enzyme inactivation and protecting the phage from restriction (Atanasiu et al. 2002; Safari et al. 2020).

Phages also manipulate bacterial modification enzymes to safeguard their genomes. Some phages acquire methyltransferases (MTases) through horizontal gene transfer, allowing them to methylate their DNA and evade restriction enzymes. For example, the *Lactococcus lactis* phage Φ50 acquired a type II MTase through recombination with a resident plasmid, providing broad resistance to bacterial R–M systems. Additionally, phage λ encodes the *ral* gene, which enhances the activity of bacterial MTases such as EcoK and EcoB, leading to increased methylation of the phage genome and resistance to cleavage. Similarly, the Lar protein, found in a hybrid phage λ (λ reverse), not only increases MTase activity but also inhibits restriction enzyme activity(Hill et al. 1991). Some phages directly degrade essential R–M cofactors to disable bacterial defenses. For example, coliphage T3 produces S-adenosyl-L-methionine hydrolase, which destroys an essential cofactor required for restriction of enzyme function(Studier and Movva 1976). Similarly, the Stp protein of phage T4 induces conformational changes in the REase EcoprrI, rendering it inactive(Penner et al. 1995).Certain phages encode their own MTases, which function independently of bacterial enzymes. Phage SPR, for example, encodes an MTase that methylates cytosines at three distinct recognition sites: GGCC, CCGG, and C(A/T)GG. Phage Mu employs a different strategy by encoding the methyl-carbamoylase Mom, which attaches an acetamide group to adenine at specific recognition sites, further protecting the phage genome from restriction (Pósfai et al. 1984).

Despite these sophisticated counter-defenses, bacteria have evolved second-line strategies to counteract anti-R–M proteins. When restriction mechanisms fail, bacteria can trigger growth arrest or programmed cell death to prevent phage replication. DNA adenine methylase (Dam), an anti-R–M protein, is a key activator of bacterial defense systems such as retron-Sen2, which induces cell growth arrest. Additionally, Dam can activate other bacterial defense mechanisms, including Dazbog and Nhi-like systems (Blower et al. 2012).

### Anti-abortive infection system

Abortive infection (Abi) is a bacterial defense mechanism that inhibits key phases of a phage’s life cycle, such as replication, transcription, and virion assembly. Abi frequently causes the death of phages and infected bacterial cells, in contrast to other resistance strategies.

All Abi systems require a toxic protein that becomes active upon phage infection, even though all Abi systems function differently. Endoribonucleases, ion channel disruptors, and transcriptional repressors are among the proteins that either inhibit bacterial growth or cause cell death, which stops phage reproduction. However, phages have developed strategies to overcome these barriers. For example, phage bIL66 suppresses the ORF3 protein, an essential enzyme for phage growth, by producing the ORF1 protein, which sets off the AbiD1 system. To bypass this, the phage mutates ORF1, preventing AbiD1 activation (Bondy-Denomy et al. 2013). Similarly, *Lactococcus* phages evade the AbiK system through mutations in *sak* genes, which encode DNA repair proteins. To resist the AbiV system, phages acquire mutations in *sav*, a gene that normally interacts with AbiV to shut down protein synthesis(Haaber et al. 2010; Scaltriti et al. 2011).Additionally, research on three *T4*-like phages revealed that they evolved spontaneous escape mutants to overcome ToxINPa-mediated abortive infection. These mutants avoid cell death through three different strategies: mutation of *asiA*, a transcriptional coactivator; alteration of *orf84*, a conserved gene; or deletion of a large Sect.  (6.5–10 kb) of their genome(Chen et al. 2017).In a more recent discovery, the phage FTE was found to counteract the ToxIN Abi system in *Pectobacterium atrosepticum* by producing an RNA molecule that mimics an antitoxin, effectively neutralizing the toxic protein ToxN. This demonstrates that the evolutionary battle between bacteria and phages is ongoing, with each side constantly developing new strategies to outmaneuver the other (Blower et al. 2012).

## Discussion

Bacterial defense mechanisms against bacteriophages are a test of the evolutionary ingenuity of microbial life. These defenses encompass a wide spectrum, ranging from surface modifications to adaptive immunity. Surface-based mechanisms such as receptor alteration, capsule formation, and biofilm production act as the first line of defense, preventing phage adsorption and penetration. Intracellular systems, including restriction-modification systems and abortive infection pathways, provide additional layers of protection by neutralizing or sacrificing infected cells. Adaptive mechanisms such as CRISPR‒Cas systems not only offer memory-based immunity but also reflect the ability of bacteria to evolve in response to repeated phage exposure. Despite these robust defenses, phages have developed equally sophisticated countermeasures, such as anti-CRISPR proteins, receptor mimicry, and depolymerases, leading to a dynamic evolutionary arms race between bacteria and phages. This interplay has profound implications for microbial ecology, evolution, and therapeutic applications. However, gaps in understanding remain, particularly concerning how bacteria coordinate multiple defenses and how environmental factors influence bacterial‒phage coevolution.

## Conclusion and future perspective

The complex interactions between bacteria and phages underscore the intricacies of microbial survival strategies. While bacterial defense mechanisms highlight their resilience, phages continue to adapt, ensuring a balance in their ongoing evolutionary struggle. These insights not only enhance our understanding of bacterial biology but also open avenues for novel antimicrobial approaches. Phage therapy, which relies on leveraging phages to combat bacterial infections, particularly multidrug-resistant strains, holds considerable promise. However, the effectiveness of this approach depends on overcoming bacterial defenses and understanding the co-evolutionary dynamics at play. Integrating molecular, ecological, and clinical perspectives is essential for unlocking the full potential of phages in modern medicine.

The future of bacterial–phage research hinges on closing current knowledge gaps and translating detailed mechanistic insights into practical therapeutic and biotechnological applications. Despite remarkable progress, challenges persist at the molecular, translational, and regulatory levels. At the molecular scale, our understanding of bacterial defense diversity and dynamics remains incomplete. Bacteria employ multi-tiered barriers against phages: outer defenses like receptor modifications and capsular variability block adsorption in *P. aeruginosa* phages; middle layers including restriction–modification systems and CRISPR–Cas immunity—among > 250 total systems including discoveries like Vibrio integron gene clusters (9 genes), Dionysus (TerB toxin), and 7 new environmental retrons (Getz et al. 2025; Table 1), degrade or neutralize invading DNA, with CRISPR acquiring protective spacers within hours; and inner mechanisms, such as abortive infection via toxin–antitoxin systems, halt replication post-entry. Biofilm formation further complicates phage action, reducing penetration, persistence, and efficacy extracellular matrices and persister cells, underscoring the need for advanced degradation and penetration studies. Long-term observational studies in natural (e.g., wastewater) and clinical (e.g., cystic fibrosis lungs) settings are essential to capture co-evolutionary dynamics, where resistance can emerge at frequencies up to 10⁶-fold, informing prediction models and adaptive intervention strategies.

From a translational perspective, phage therapy faces significant hurdles. Narrow host ranges (typically 1–5 bacterial strains per phage), rapid bacterial resistance evolution (7–14 days in vitro), and inconsistent in vivo efficacy (20–80% variability) limit therapeutic reliability, particularly against multidrug-resistant pathogens such as *Klebsiella pneumoniae* and *Acinetobacter baumannii*. Rational phage engineering offers solutions by enhancing infectivity through tail fiber shuffling (expanding host range 5–10×), incorporating anti-CRISPR proteins to bypass immunity, and stabilizing genomes for improved persistence. Combination approaches, which pair phages with antibiotics (restoring sensitivity in up to 90% of MDR cases), biofilm-disrupting enzymes such as DNase, or quorum-sensing inhibitors, provide synergistic effects. Integration with nanomedicine, such as PEGylated liposomal dry powder formulations for pulmonary delivery, can enhance lung retention by 3–5 times, evade immune clearance, and enable treatments tailored to specific pathogens, infection sites, and patient immune profiles.

Beyond technical considerations, regulatory, ethical, and safety challenges must be addressed. Regulatory efforts should focus on GMP-compliant production and pharmacovigilance frameworks. Ethical considerations include the ecological impact of phage release and ensuring equitable global access. Safety concerns require monitoring for immune reactions and off-target effects. Establishing clear guidelines, standardized clinical protocols, and compassionate-use pathways (e.g., FDA expansions) will be critical for responsible adoption. Importantly, these defense strategies are not universally advantageous and involve clear evolutionary trade-offs (Anomaly 2020). Surface modifications and capsule loss reduce phage adsorption but often compromise virulence and environmental fitness, whereas intracellular systems such as abortive infection provide population-level protection at the cost of individual cell survival. CRISPR–Cas immunity offers adaptive and heritable defense, yet imposes metabolic costs and risks autoimmunity, making it less favorable under high horizontal gene transfer pressures. These contrasts suggest that no single defense strategy is optimal across environments; instead, bacteria dynamically balance specificity, cost, and durability in response to phage predation. A critical challenge for the field is resolving how these trade-offs shape long-term evolutionary stability across ecological contexts. By combining rigorous mechanistic research, informed by genomics and AI-based predictive modeling, with innovative translational strategies, bacterial–phage studies can reshape the fight against antimicrobial resistance and advance management of microbial ecosystems on a global scale.

## Data Availability

No datasets were generated or analyzed during the current study.

## References

[CR1] Abd-Allah IM, El-Housseiny GS, Yahia IS, Aboshanab KM, Hassouna NA (2021) Rekindling of a masterful precedent; Bacteriophage: Reappraisal and future pursuits. Front Cell Infect Microbiol 11:635597 34136415 10.3389/fcimb.2021.635597 PMC8201069

[CR2] Abedon ST (2011) Lysis from without. Bacteriophage 1(1):46–49 21687534 10.4161/bact.1.1.13980 PMC3109453

[CR3] Aframian N, Eldar A (2023) Abortive infection antiphage defense systems: separating mechanism and phenotype. Trends Microbiol 31(10):1003–1012 37268559 10.1016/j.tim.2023.05.002

[CR4] Ahmed MM, Kayode HH, Okesanya OJ, Ukoaka BM, Eshun G, Mourid MR, Adigun OA, Ogaya JB, Mohamed ZO, Lucero-Prisno III, D. E (2024) CRISPR-Cas systems in the fight against antimicrobial resistance: current status, potentials, and future directions. Infect Drug Resist, 5229–5245 10.2147/IDR.S494327 PMC11608035 39619730

[CR5] Anastassopoulou C, Tsakri D, Panagiotopoulos A-P, Saldari C, Sagona AP, Tsakris A (2025) Armed phages: a New Weapon in the battle against antimicrobial resistance. Viruses 17(7):911 40733529 10.3390/v17070911 PMC12300627

[CR6] Anomaly J (2020) The Future of Phage: Ethical challenges of using phage viruses to treat bacterial infections 10.1093/phe/phaa003 PMC7392637 32760449

[CR7] Atanasiu C, Su T-J, Sturrock SS, Dryden DTF (2002) Interaction of the ocr gene 0.3 protein of bacteriophage T7 with Eco KI restriction/modification enzyme. Nucleic Acids Res 30(18):3936–3944 12235377 10.1093/nar/gkf518 PMC137103

[CR8] Athukoralage JS, White MF (2022) Cyclic nucleotide signaling in phage defense and counter-defense. Annual Rev Virol 9(1):451–468 35567297 10.1146/annurev-virology-100120-010228

[CR9] Baptista C, Santos MA, Sao-José C (2008) Phage SPP1 reversible adsorption to Bacillus subtilis cell wall teichoic acids accelerates virus recognition of membrane receptor YueB. J Bacteriol 190(14):4989–4996 18487323 10.1128/JB.00349-08 PMC2446999

[CR11] Barrangou R, Marraffini LA (2014) CRISPR-cas systems: prokaryotes upgrade to adaptive immunity. Mol Cell 54(2):234–244 24766887 10.1016/j.molcel.2014.03.011 PMC4025954

[CR10] Barrangou R, Fremaux C, Deveau H, Richards M, Boyaval P, Moineau S, Romero DA, Horvath P (2007) CRISPR provides acquired resistance against viruses in prokaryotes. Science 315(5819):1709–1712 17379808 10.1126/science.1138140

[CR12] Bebeacua C, Tremblay D, Farenc C, Chapot-Chartier M-P, Sadovskaya I, Van Heel M, Veesler D, Moineau S, Cambillau C (2013) Structure, adsorption to host, and infection mechanism of virulent lactococcal phage p2. J Virol 87(22):12302–12312 24027307 10.1128/JVI.02033-13 PMC3807928

[CR13] Beck IN, Picton DM, Blower TR (2022) Crystal structure of the BREX phage defence protein BrxA. Curr Res Struct Biology 4:211–219 10.1016/j.crstbi.2022.06.001 PMC9240713 35783086

[CR14] Benler S, Cobián-Güemes AG, McNair K, Hung S-H, Levi K, Edwards R, Rohwer F (2018) A diversity-generating retroelement encoded by a globally ubiquitous Bacteroides phage. Microbiome 6:1–10 30352623 10.1186/s40168-018-0573-6 PMC6199706

[CR16] Bernheim A, Sorek R (2020) The pan-immune system of bacteria: antiviral defence as a community resource. Nat Rev Microbiol 18(2):113–119 31695182 10.1038/s41579-019-0278-2

[CR15] Bernheim A, Millman A, Ofir G, Meitav G, Avraham C, Shomar H, Rosenberg MM, Tal N, Melamed S, Amitai G, others (2021) Prokaryotic viperins produce diverse antiviral molecules. Nature 589(7840):120–124 32937646 10.1038/s41586-020-2762-2 PMC7610908

[CR17] Bertozzi Silva J, Storms Z, Sauvageau D (2016) Host receptors for bacteriophage adsorption. FEMS Microbiol Lett 363(4):fnw002 26755501 10.1093/femsle/fnw002

[CR18] Bishop RE (2008) Structural biology of membrane-intrinsic $β$-barrel enzymes: Sentinels of the bacterial outer membrane. Biochim et Biophys Acta (BBA)-Biomembranes 1778(9):1881–1896 10.1016/j.bbamem.2007.07.021 PMC5007122 17880914

[CR19] Blokesch M (2025) Defence systems encoded by core genomic islands of seventh pandemic Vibrio cholerae. *Philosophical Transactions of the Royal Society B: Biological Sciences*, *380*(1934) 10.1098/rstb.2024.0083 PMC12409362 40904118

[CR20] Blower TR, Evans TJ, Przybilski R, Fineran PC, Salmond GPC (2012) Viral evasion of a bacterial suicide system by RNA–based molecular mimicry enables infectious altruism 10.1371/journal.pgen.1003023 PMC3475682 23109916

[CR21] Bondy-Denomy J, Pawluk A, Maxwell KL, Davidson AR (2013) Bacteriophage genes that inactivate the CRISPR/Cas bacterial immune system. Nature 493(7432):429–432 23242138 10.1038/nature11723 PMC4931913

[CR22] Bravo JPK, Aparicio-Maldonado C, Nobrega FL, Brouns SJJ, Taylor DW (2022) Structural basis for broad anti-phage immunity by DISARM. Nat Commun 13(1):2987 35624106 10.1038/s41467-022-30673-1 PMC9142583

[CR23] Breitbart M, Rohwer F (2005) Here a virus, there a virus, everywhere the same virus? Trends Microbiol 13(6):278–284 15936660 10.1016/j.tim.2005.04.003

[CR24] Brown S, Santa Maria JP Jr, Walker S (2013) Wall teichoic acids of gram-positive bacteria. Annu Rev Microbiol 67(1):313–336 24024634 10.1146/annurev-micro-092412-155620 PMC3883102

[CR25] Brüssow H, Canchaya C, Hardt W-D (2004) Phages and the evolution of bacterial pathogens: from genomic rearrangements to lysogenic conversion. Microbiol Mol Biol Rev 68(3):560–602 15353570 10.1128/MMBR.68.3.560-602.2004 PMC515249

[CR26] Bucher MJ, Czyż DM (2024) Phage against the machine: The SIE-ence of superinfection exclusion. Viruses 16(9):1348 39339825 10.3390/v16091348 PMC11436027

[CR27] Camara-Wilpert S, Mayo-Muñoz D, Russel J, Fagerlund RD, Madsen JS, Fineran PC, Sørensen SJ, Pinilla-Redondo R (2023) Bacteriophages suppress CRISPR–Cas immunity using RNA-based anti-CRISPRs. Nature 623(7987):601–607 37853129 10.1038/s41586-023-06612-5 PMC10651486

[CR28] Caroff M, Novikov A (2019) LPS structure, function, and heterogeneity. Endotoxin Detect Control Pharma Limulus Mammalian Syst, 53–93

[CR29] Carroll-Portillo A, Lin HC (2019) Bacteriophage and the innate immune system: access and signaling. Microorganisms 7(12):625 31795262 10.3390/microorganisms7120625 PMC6956183

[CR30] Carvalho F, Sousa S, Cabanes D (2014) How Listeria monocytogenes organizes its surface for virulence. Front Cell Infect Microbiol 4:48 24809022 10.3389/fcimb.2014.00048 PMC4010754

[CR31] Chatterjee S, Rothenberg E (2012) Interaction of bacteriophage $λ$ with its E. coli receptor, LamB. Viruses 4(11):3162–3178 23202520 10.3390/v4113162 PMC3509688

[CR32] Chen B, Akusobi C, Fang X, Salmond GPC (2017) Environmental T4-family bacteriophages evolve to escape abortive infection via multiple routes in a bacterial host employing altruistic suicide through type III toxin-antitoxin systems. Front Microbiol 8:1006 28620370 10.3389/fmicb.2017.01006 PMC5449768

[CR33] Chopin M-C, Chopin A, Bidnenko E (2005) Phage abortive infection in lactococci: variations on a theme. Curr Opin Microbiol 8(4):473–479 15979388 10.1016/j.mib.2005.06.006

[CR35] Clokie MR, Kropinski A (2009) Methods and protocols, 1: Isolation, characterization, and interactions. Methods Mol Biology Humana Press, 69–81

[CR34] Clokie MRJ, Millard AD, Letarov AV, Heaphy S (2011) Phages in nature. Bacteriophage 1(1):31–45 21687533 10.4161/bact.1.1.14942 PMC3109452

[CR36] Cornelissen A, Ceyssens P-J, Krylov VN, Noben J-P, Volckaert G, Lavigne R (2012) Identification of EPS-degrading activity within the tail spikes of the novel Pseudomonas putida phage AF. Virology 434(2):251–256 23084421 10.1016/j.virol.2012.09.030

[CR37] Cota I, Sánchez-Romero MA, Hernández SB, Pucciarelli MG, Garc\’\ia-Del Portillo, F., Casadesús J (2015) Epigenetic control of Salmonella enterica O-antigen chain length: a tradeoff between virulence and bacteriophage resistance. *PLoS Genetics*, *11*(11), e1005667 10.1371/journal.pgen.1005667 PMC4652898 26583926

[CR38] de Jonge PA, Nobrega FL, Brouns SJJ, Dutilh BE (2019) Molecular and evolutionary determinants of bacteriophage host range. Trends Microbiol 27(1):51–63 30181062 10.1016/j.tim.2018.08.006

[CR39] Debiasi-Anders G, Qiao C, Salim A, Li N, Mir-Sanchis I (2025) Phage parasites targeting phage homologous recombinases provide antiviral immunity. Nat Commun 16(1):1889 39987160 10.1038/s41467-025-57156-3 PMC11846896

[CR40] Denes T, den Bakker HC, Tokman JI, Guldimann C, Wiedmann M (2015) Selection and characterization of phage-resistant mutant strains of Listeria monocytogenes reveal host genes linked to phage adsorption. Appl Environ Microbiol 81(13):4295–4305 25888172 10.1128/AEM.00087-15 PMC4475870

[CR41] Dennehy JJ, Abedon ST (2021) Phage infection and lysis. Bacteriophages: Biology Technol Therapy, 341–383

[CR42] Dicks LMT, Vermeulen W (2024) Bacteriophage–Host Interactions and the Therapeutic Potential of Bacteriophages. Viruses 16(3):478 38543843 10.3390/v16030478 PMC10975011

[CR43] Ding Y, Zhang D, Zhao X, Tan W, Zheng X, Zhang Q, Ji X, Wei Y (2021) Autoinducer-2-mediated quorum-sensing system resists T4 phage infection in Escherichia coli. J Basic Microbiol 61(12):1113–1123 34783039 10.1002/jobm.202100344

[CR44] Dixon DR, Darveau RP (2005) Lipopolysaccharide heterogeneity: innate host responses to bacterial modification of lipid a structure. J Dent Res 84(7):584–595 15972584 10.1177/154405910508400702

[CR45] Doron S, Melamed S, Ofir G, Leavitt A, Lopatina A, Keren M, Amitai G, Sorek R (2018) Systematic discovery of antiphage defense systems in the microbial pangenome. Science 359(6379):eaar4120 29371424 10.1126/science.aar4120 PMC6387622

[CR46] Dot EW, Thomason LC, Chappie JS (2023) Everything OLD is new again: How structural, functional, and bioinformatic advances have redefined a neglected nuclease family. Mol Microbiol 120(2):122–140 37254295 10.1111/mmi.15074

[CR47] Drozdz M, Piekarowicz A, Bujnicki JM, Radlinska M (2012) Novel non-specific DNA adenine methyltransferases. Nucleic Acids Res 40(5):2119–2130 22102579 10.1093/nar/gkr1039 PMC3299994

[CR48] Du A (2023) The virus resistance mechanism of abortive infection K in lactococcus lactis. Virus, 7

[CR50] Dunne M, Rupf B, Tala M, Qabrati X, Ernst P, Shen Y, Sumrall E, Heeb L, Plückthun A, Loessner MJ (2019) & others. Reprogramming bacteriophage host range through structure-guided design of chimeric receptor binding proteins. *Cell Reports*, *29*(5), 1336–1350 10.1016/j.celrep.2019.09.062 31665644

[CR49] Dunne M, Prokhorov NS, Loessner MJ, Leiman PG (2021) Reprogramming bacteriophage host range: design principles and strategies for engineering receptor binding proteins. Curr Opin Biotechnol 68:272–281 33744824 10.1016/j.copbio.2021.02.006 PMC10163921

[CR51] Dunsing V, Irmscher T, Barbirz S, Chiantia S (2019) Purely polysaccharide-based biofilm matrix provides size-selective diffusion barriers for nanoparticles and bacteriophages. Biomacromolecules 20(10):3842–3854 31478651 10.1021/acs.biomac.9b00938

[CR52] Dy RL, Richter C, Salmond GPC, Fineran PC (2014) Remarkable mechanisms in microbes to resist phage infections. Annual Rev Virol 1(1):307–331 26958724 10.1146/annurev-virology-031413-085500

[CR53] Engelberg-Kulka H, Amitai S, Kolodkin-Gal I, Hazan R (2006) Bacterial programmed cell death and multicellular behavior in bacteria. PLoS Genet, 2(10), e135 10.1371/journal.pgen.0020135 PMC1626106 17069462

[CR54] Esteves NC, Scharf BE (2022) Flagellotropic bacteriophages: opportunities and challenges for antimicrobial applications. Int J Mol Sci 23(13):7084 35806089 10.3390/ijms23137084 PMC9266447

[CR55] Fehmel F, Feige U, Niemann H, Stirm S (1975) Escherichia coli capsule bacteriophages. VII. Bacteriophage 29-host capsular polysaccharide interactions. J Virol 16(3):591–601 1099233 10.1128/jvi.16.3.591-601.1975 PMC354707

[CR56] Fernandes S, São-José C (2018) Enzymes and mechanisms employed by tailed bacteriophages to breach the bacterial cell barriers. Viruses 10(8):396 30060520 10.3390/v10080396 PMC6116005

[CR57] Fernández L, Rodr\’\iguez A, Garc\’\ia P (2018) Phage or foe: an insight into the impact of viral predation on microbial communities. ISME J 12(5):1171–1179 29371652 10.1038/s41396-018-0049-5 PMC5932045

[CR58] Ferriol-González C, Domingo-Calap P (2020) Phages for biofilm removal. Antibiotics 9(5):268 32455536 10.3390/antibiotics9050268 PMC7277876

[CR59] Fineran PC, Blower TR, Foulds IJ, Humphreys DP, Lilley KS, Salmond GPC (2009) The phage abortive infection system, ToxIN, functions as a protein–RNA toxin–antitoxin pair. *Proceedings of the National Academy of Sciences*, *106*(3), 894–899 10.1073/pnas.0808832106 PMC2630095 19124776

[CR60] Gao L, Altae-Tran H, Böhning F, Makarova KS, Segel M, Schmid-Burgk JL, Koob J, Wolf YI, Koonin EV, Zhang F (2020) Diverse enzymatic activities mediate antiviral immunity in prokaryotes. Science 369(6507):1077–1084 32855333 10.1126/science.aba0372 PMC7985843

[CR61] Garb J, Lopatina A, Bernheim A, Zaremba M, Siksnys V, Melamed S, Leavitt A, Millman A, Amitai G, Sorek R (2022) Multiple phage resistance systems inhibit infection via SIR2-dependent NAD+ depletion. Nat Microbiol 7(11):1849–1856 36192536 10.1038/s41564-022-01207-8

[CR62] Garen A, Puck TT (1951) The first two steps of the invasion of host cells by bacterial viruses. II. J Exp Med 94(3):177 14861377 10.1084/jem.94.3.177 PMC2136106

[CR63] Garneau JE, Dupuis M-È, Villion M, Romero DA, Barrangou R, Boyaval P, Fremaux C, Horvath P, Magadán AH, Moineau S (2010) The CRISPR/Cas bacterial immune system cleaves bacteriophage and plasmid DNA. Nature 468(7320):67–71 21048762 10.1038/nature09523

[CR64] Gasperini G, Massai L, De Simone D, Raso MM, Palmieri E, Alfini R, Rossi O, Ravenscroft N, Kuttel MM, Micoli F (2024) O-Antigen decorations in Salmonella enterica play a key role in eliciting functional immune responses against heterologous serovars in animal models. Front Cell Infect Microbiol 14:1347813 38487353 10.3389/fcimb.2024.1347813 PMC10937413

[CR65] Ge Y, Gao A, Zhu Y (2025) Molecular mechanisms of CBASS-mediated bacteriophage defense. Biophys Rep

[CR66] Getz LJ, Fairburn SR, Liu V, Qian Y, A. L., Maxwell KL (2025) Integrons are anti-phage defence libraries in Vibrio parahaemolyticus. Nat Microbiol 10(3):724–733 39870871 10.1038/s41564-025-01927-7

[CR67] Giordano NP, Cian MB, Dalebroux ZD (2020) Outer membrane lipid secretion and the innate immune response to gram-negative bacteria. Infect Immun 88(7):10–1128 10.1128/IAI.00920-19 PMC7309610 32253250

[CR68] Goldfarb T, Sberro H, Weinstock E, Cohen O, Doron S, Charpak-Amikam Y, Afik S, Ofir G, Sorek R (2015) BREX is a novel phage resistance system widespread in microbial genomes. EMBO J 34(2):169–183 25452498 10.15252/embj.201489455 PMC4337064

[CR69] Gordillo Altamirano FL, Barr JJ (2019) Phage therapy in the postantibiotic era. Clin Microbiol Rev 32(2):10–1128 10.1128/CMR.00066-18 PMC6431132 30651225

[CR70] Guegler CK, Laub MT (2021) Shutoff of host transcription triggers a toxin-antitoxin system to cleave phage RNA and abort infection. Mol Cell 81(11):2361–2373 33838104 10.1016/j.molcel.2021.03.027 PMC8284924

[CR71] Guerrero-Ferreira RC, Viollier PH, Ely B, Poindexter JS, Georgieva M, Jensen GJ, Wright ER (2011) Alternative mechanism for bacteriophage adsorption to the motile bacterium Caulobacter crescentus. Proc Natl Acad Sci 108(24):9963–9968 21613567 10.1073/pnas.1012388108 PMC3116389

[CR72] Haaber J, Samson JE, Labrie SJ, Campanacci V, Cambillau C, Moineau S, Hammer K (2010) Lactococcal abortive infection protein AbiV interacts directly with the phage protein SaV and prevents translation of phage proteins. Appl Environ Microbiol 76(21):7085–7092 20851990 10.1128/AEM.00093-10 PMC2976256

[CR73] Hanlon GW, Denyer SP, Olliff CJ, Ibrahim LJ (2001) Reduction in exopolysaccharide viscosity as an aid to bacteriophage penetration through Pseudomonas aeruginosa biofilms. Appl Environ Microbiol 67(6):2746–2753 11375190 10.1128/AEM.67.6.2746-2753.2001 PMC92934

[CR74] Harper DR, Parracho H, Walker J, Sharp R, Hughes G, Werthén M, Lehman S, Morales S (2014) Bacteriophages and biofilms. Antibiotics 3 (3): 270–284

[CR75] Hasan M, Ahn J (2022) Evolutionary dynamics between phages and bacteria as a possible approach for designing effective phage therapies against antibiotic-resistant bacteria. Antibiotics 11(7):915 35884169 10.3390/antibiotics11070915 PMC9311878

[CR76] Hays SG, Seed KD (2020) Dominant Vibrio cholerae phage exhibits lysis inhibition sensitive to disruption by a defensive phage satellite. Elife 9:e53200 32329714 10.7554/eLife.53200 PMC7182436

[CR77] Heller K, Braun V (1982) Polymannose O-antigens of Escherichia coli, the binding sites for the reversible adsorption of bacteriophage T5 + via the L-shaped tail fibers. J Virol 41(1):222–227 7045389 10.1128/jvi.41.1.222-227.1982 PMC256742

[CR78] Henderson B, Poole S, Wilson M (1996) Bacterial modulins: a novel class of virulence factors which cause host tissue pathology by inducing cytokine synthesis. Microbiol Rev 60(2):316–341 8801436 10.1128/mr.60.2.316-341.1996 PMC239446

[CR79] Hill C, Miller LA, Klaenhammer TR (1991) In vivo genetic exchange of a functional domain from a type II A methylase between lactococcal plasmid pTR2030 and a virulent bacteriophage. J Bacteriol 173(14):4363–4370 1906061 10.1128/jb.173.14.4363-4370.1991 PMC208097

[CR80] Hille F, Richter H, Wong SP, Bratovič M, Ressel S, Charpentier E (2018) The biology of CRISPR-Cas: backward and forward. Cell 172(6):1239–1259 29522745 10.1016/j.cell.2017.11.032

[CR81] Høyland-Kroghsbo NM, Mærkedahl RB, Svenningsen S, Lo (2013) A quorum-sensing-induced bacteriophage defense mechanism. MBio 4(1):10–1128 10.1128/mBio.00362-12 PMC3624510 23422409

[CR82] Hryhorowicz M, Lipiński D, Zeyland J (2023) Evolution of CRISPR/cas systems for precise genome editing. Int J Mol Sci 24(18):14233 37762535 10.3390/ijms241814233 PMC10532350

[CR83] Hsu PD, Lander ES, Zhang F (2014) Development and applications of CRISPR-Cas9 for genome engineering. Cell 157(6):1262–1278 24906146 10.1016/j.cell.2014.05.010 PMC4343198

[CR84] Hyman P, Abedon ST (2010) Bacteriophage host range and bacterial resistance. Adv Appl Microbiol 70:217–248 20359459 10.1016/S0065-2164(10)70007-1

[CR85] Iida S, Streiff MB, Bickle TA, Arber W (1987) Two DNA antirestriction systems of bacteriophage P1, darA, and darB: characterization of darA- phages. Virology 157(1):156–166 3029954 10.1016/0042-6822(87)90324-2

[CR86] Isaev AB, Musharova OS, Severinov KV (2021) Microbial arsenal of antiviral defenses–part I. Biochem (Moscow) 86(3):319–337 10.1134/S0006297921030081 33838632

[CR87] Izano EA, Amarante MA, Kher WB, Kaplan JB (2008) Differential roles of poly-N-acetylglucosamine surface polysaccharide and extracellular DNA in Staphylococcus aureus and Staphylococcus epidermidis biofilms. Appl Environ Microbiol 74(2):470–476 18039822 10.1128/AEM.02073-07 PMC2223269

[CR89] Jiang Z, Wei J, Liang Y, Peng N, Li Y (2020) Aminoglycoside antibiotics inhibit mycobacteriophage infection. Antibiotics 9(10):714 33086520 10.3390/antibiotics9100714 PMC7603143

[CR88] Jiang S, Chen K, Wang Y, Zhang Y, Tang Y, Huang W, Xiong X, Chen S, Chen C, Wang L (2023) A DNA phosphorothioation-based Dnd defense system provides resistance against various phages and is compatible with the Ssp defense system. Mbio 14(4):e00933–e00923 37260233 10.1128/mbio.00933-23 PMC10470545

[CR90] Johnson AG, Kranzusch PJ (2022) What bacterial cell death teaches us about life. PLoS Pathog, 18(10), e1010879 10.1371/journal.ppat.1010879 PMC9612521 36301823

[CR91] Johnson MC, Laderman E, Huiting E, Zhang C, Davidson A, Bondy-Denomy J (2023) Core defense hotspots within Pseudomonas aeruginosa are a consistent and rich source of anti-phage defense systems. Nucleic Acids Res 51(10):4995–5005 37140042 10.1093/nar/gkad317 PMC10250203

[CR92] Juszczuk-Kubiak E (2024) Molecular aspects of the functioning of pathogenic bacteria biofilm based on Quorum Sensing (QS) signal-response system and innovative non-antibiotic strategies for their elimination. Int J Mol Sci 25(5):2655 38473900 10.3390/ijms25052655 PMC10931677

[CR93] Karygianni L, Ren Z, Koo H, Thurnheer T (2020) Biofilm matrixome: extracellular components in structured microbial communities. Trends Microbiol 28(8):668–681 32663461 10.1016/j.tim.2020.03.016

[CR94] Kaufmann G (2000) Anticodon nucleases. Trends Biochem Sci 25(2):70–74 10664586 10.1016/s0968-0004(99)01525-x

[CR95] Knirel YA, Shneider MM, Popova AV, Kasimova AA, Senchenkova SN, Shashkov AS, Chizhov AO (2020) Mechanisms of Acinetobacter baumannii capsular polysaccharide cleavage by phage depolymerases. Biochem (Moscow) 85:567–574 10.1134/S0006297920050053 32571186

[CR96] Koonin EV, Makarova KS, Zhang F (2017) Diversity, classification and evolution of CRISPR-Cas systems. Curr Opin Microbiol 37:67–78 28605718 10.1016/j.mib.2017.05.008 PMC5776717

[CR97] Korn D (1967) Inhibition of bacteriophage T4 deoxyribonucleic acid maturation by actinomycin D: The accumulation of replicating deoxyribonucleic acid. J Biol Chem 242(1):160–162 6016329

[CR98] Kronheim S, Daniel-Ivad M, Duan Z, Hwang S, Wong AI, Mantel I, Nodwell JR, Maxwell KL (2018) A chemical defence against phage infection. Nature 564(7735):283–286 30518855 10.1038/s41586-018-0767-x

[CR99] Krüger DH, Bickle TA (1983) Bacteriophage survival: multiple mechanisms for avoiding the deoxyribonucleic acid restriction systems of their hosts. Microbiol Rev 47(3):345–360 6314109 10.1128/mr.47.3.345-360.1983 PMC281580

[CR100] Labrie SJ, Samson JE, Moineau S (2010) Bacteriophage resistance mechanisms. Nat Rev Microbiol 8(5):317–327 20348932 10.1038/nrmicro2315

[CR101] Le KY, Park MD, Otto M (2018) Immune evasion mechanisms of Staphylococcus epidermidis biofilm infection. Front Microbiol 9:359 29541068 10.3389/fmicb.2018.00359 PMC5835508

[CR102] León M, Bast\’\ias R (2015) Virulence reduction in bacteriophage resistant bacteria. Front Microbiol 6:343 25954266 10.3389/fmicb.2015.00343 PMC4407575

[CR103] Leprince A, Mahillon J (2023) Phage adsorption to gram-positive bacteria. Viruses 15(1):196 36680236 10.3390/v15010196 PMC9863714

[CR104] Li C, Alam K, Zhao Y, Hao J, Yang Q, Zhang Y, Li R, Li A (2021) Mining and biosynthesis of bioactive lanthipeptides from microorganisms. Front Bioeng Biotechnol 9:692466 34395400 10.3389/fbioe.2021.692466 PMC8358304

[CR105] Li S, Xu T, Meng X, Yan Y, Zhou Y, Duan L, Tang Y, Zhu L, Sun L (2024) Ocr-mediated suppression of BrxX unveils a phage counter-defense mechanism. Nucleic Acids Res 52(14):8580–8594 38989624 10.1093/nar/gkae608 PMC11317158

[CR106] Loenen WAM, Dryden DTF, Raleigh EA, Wilson GG, Murray NE (2014) Highlights of the DNA cutters: a short history of the restriction enzymes. Nucleic Acids Res 42(1):3–19 24141096 10.1093/nar/gkt990 PMC3874209

[CR107] Lopatina A, Tal N, Sorek R (2020) Abortive infection: bacterial suicide as an antiviral immune strategy. Annual Rev Virol 7(1):371–384 32559405 10.1146/annurev-virology-011620-040628

[CR108] Loyo C, Grossman AD (2024) A phage-encoded counter-defense inhibits an NAD-degrading anti-phage defense system. BioRxiv, 2012–2024 10.1371/journal.pgen.1011551 PMC11984713 40173202

[CR109] Madsen JS, Burmølle M, Hansen LH, Sørensen SJ (2012) The interconnection between biofilm formation and horizontal gene transfer. FEMS Immunol \& Med Microbiol 65(2):183–195 22444301 10.1111/j.1574-695X.2012.00960.x

[CR110] Mahony J, McDonnell B, Casey E, van Sinderen D (2016) Phage-host interactions of cheese-making lactic acid bacteria. Annual Rev Food Sci Technol 7(1):267–285 26735798 10.1146/annurev-food-041715-033322

[CR113] Makarova KS, Wolf YI, Snir S, Koonin EV (2011) Defense islands in bacterial and archaeal genomes and prediction of novel defense systems. J Bacteriol 193(21):6039–6056 21908672 10.1128/JB.05535-11 PMC3194920

[CR112] Makarova KS, Wolf YI, Iranzo J, Shmakov SA, Alkhnbashi OS, Brouns SJJ, Charpentier E, Cheng D, Haft DH, Horvath P, others (2020) Evolutionary classification of CRISPR–Cas systems: a burst of class 2 and derived variants. Nat Rev Microbiol 18(2):67–83 31857715 10.1038/s41579-019-0299-x PMC8905525

[CR111] Makarova KS, Shmakov SA, Wolf YI, Mutz P, Altae-Tran H, Beisel CL, Brouns SJJ, Charpentier E, Cheng D, Doudna J (2025) & others. An updated evolutionary classification of CRISPR–Cas systems including rare variants. *Nature Microbiology*, 1–16 10.1038/s41564-025-02180-8 PMC12669027 41198952

[CR114] Marti R, Zurfluh K, Hagens S, Pianezzi J, Klumpp J, Loessner MJ (2013) Long tail fibres of the novel broad-host-range T-even bacteriophage S 16 specifically recognize S almonella OmpC. Mol Microbiol 87(4):818–834 23289425 10.1111/mmi.12134

[CR115] Millman A, Bernheim A, Stokar-Avihail A, Fedorenko T, Voichek M, Leavitt A, Oppenheimer-Shaanan Y, Sorek R (2020) Bacterial retrons function in anti-phage defense. Cell 183(6):1551–1561 33157039 10.1016/j.cell.2020.09.065

[CR116] Millman A, Melamed S, Leavitt A, Doron S, Bernheim A, Hör J, Garb J, Bechon N, Brandis A, Lopatina A (2022) & others. An expanded arsenal of immune systems that protect bacteria from phages. *Cell Host \& Microbe*, *30*(11), 1556–1569 10.1016/j.chom.2022.09.017 36302390

[CR117] Morita J, Tanaka A, Komano T, Oki T (1979) Inactivation of phage $φ$ X174 by anthracycline antibiotics, aclacinomycin A, doxorubicin and daunorubicin. Agric Biol Chem 43(12):2629–2631

[CR118] Moskowitz SM, Ernst RK, Miller SI (2004) PmrAB, a two-component regulatory system of Pseudomonas aeruginosa that modulates resistance to cationic antimicrobial peptides and addition of aminoarabinose to lipid A. J Bacteriol 186(2):575–579 14702327 10.1128/JB.186.2.575-579.2004 PMC305751

[CR119] Munita JM, Arias CA (2016) Mechanisms of antibiotic resistance. Virulence Mech Bacterial Pathogens, 481–511

[CR120] Murray NE (2000) Type I restriction systems: sophisticated molecular machines (a legacy of Bertani and Weigle). Microbiol Mol Biol Rev 64(2):412–434 10839821 10.1128/mmbr.64.2.412-434.2000 PMC98998

[CR121] Murtazalieva K, Mu A, Petrovskaya A, Finn RD (2024) The growing repertoire of phage anti-defence systems. Trends Microbiol 10.1016/j.tim.2024.05.005 38845267

[CR122] Nahar S, Mizan MFR, Ha AJ, Ha S-D (2018) Advances and future prospects of enzyme-based biofilm prevention approaches in the food industry. Compr Rev Food Sci Food Saf 17(6):1484–1502 33350139 10.1111/1541-4337.12382

[CR123] Ngene AC, Umar U (2025) Bacteriophage applications and future prospects for addressing challenges in African agrifood systems. Discover Food 5(1):429

[CR124] Nikolic N (2019) Autoregulation of bacterial gene expression: lessons from the MazEF toxin–antitoxin system. Curr Genet 65(1):133–138 30132188 10.1007/s00294-018-0879-8 PMC6343021

[CR125] Nobrega FL, Vlot M, De Jonge PA, Dreesens LL, Beaumont HJE, Lavigne R, Dutilh BE, Brouns SJJ (2018) Targeting mechanisms of tailed bacteriophages. Nat Rev Microbiol 16(12):760–773 30104690 10.1038/s41579-018-0070-8

[CR126] Nwokolo NL, Enebe MC, Chigor CB, Pathom-aree W, Chigor VN (2024) THE IMPACTS OF PHAGE-ACTINOBACTERIAL INTERACTIONS ON THE ECOLOGICAL FUNCTIONS OF ACTINOBACTERIA. Microbe, 100042

[CR127] Oechslin F (2018) Resistance development to bacteriophages occurring during bacteriophage therapy. Viruses 10(7):351 29966329 10.3390/v10070351 PMC6070868

[CR128] Ofir G, Melamed S, Sberro H, Mukamel Z, Silverman S, Yaakov G, Doron S, Sorek R (2018) DISARM is a widespread bacterial defence system with broad anti-phage activities. Nat Microbiol 3(1):90–98 29085076 10.1038/s41564-017-0051-0 PMC5739279

[CR129] Opperman CJ, Wojno J, Goosen W, Warren R (2023) Phages for the treatment of Mycobacterium species. Prog Mol Biol Transl Sci 201:41–92 37770176 10.1016/bs.pmbts.2023.03.016

[CR130] Pan H, Lou Y, Zeng L, Wang L, Zhang J, Yu W, Qiu Y (2019a) Infections caused by carbapenemase-producing Klebsiella pneumoniae: microbiological characteristics and risk factors. Microb Drug Resist 25(2):287–296 30810470 10.1089/mdr.2018.0339 PMC6441289

[CR131] Pan Y-J, Lin T-L, Chen Y-Y, Lai P-H, Tsai Y-T, Hsu C-R, Hsieh P-F, Lin Y-T, Wang J-T (2019b) Identification of three podoviruses infecting Klebsiella encoding capsule depolymerases that digest specific capsular types. Microb Biotechnol 12(3):472–486 30706654 10.1111/1751-7915.13370 PMC6465236

[CR132] Paoli L, Laruelle B, Lavenir R, Loubat A, Tesson F, Gaborieau B, Bernheim A (2025) Environment and physiology shape antiphage system expression. BioRxiv, 2012–2025

[CR133] Paracini N, Schneck E, Imberty A, Micciulla S (2022) Lipopolysaccharides at solid and liquid interfaces: models for biophysical studies of the gram-negative bacterial outer membrane. Adv Colloid Interface Sci 301:102603 35093846 10.1016/j.cis.2022.102603

[CR134] Pawluk AIE (2016) Prevalence, Diversity, Impact, and Applications of CRISPR-Cas Inhibitors. University of Toronto (Canada)

[CR135] Penner M, Morad I, Snyder L, Kaufmann G (1995) Phage T4-coded Stp: double-edged effector of coupled DNA and tRNA-restriction systems. J Mol Biol 249(5):857–868 7791212 10.1006/jmbi.1995.0343

[CR136] Philippe C, Cornuault JK, de Melo AG, Morin-Pelchat R, Jolicoeur AP, Moineau S (2023) The never-ending battle between lactic acid bacteria and their phages. FEMS Microbiol Rev 47(4):fuad035 37353926 10.1093/femsre/fuad035

[CR137] Picton DM, Luyten YA, Morgan RD, Nelson A, Smith DL, Dryden DTF, Hinton JCD, Blower TR (2021) The phage defence island of a multidrug resistant plasmid uses both BREX and type IV restriction for complementary protection from viruses. Nucleic Acids Res 49(19):11257–11273 34657954 10.1093/nar/gkab906 PMC8565348

[CR138] Pires DP, Melo LDR, Boas DV, Sillankorva S, Azeredo J (2017) Phage therapy as an alternative or complementary strategy to prevent and control biofilm-related infections. Curr Opin Microbiol 39:48–56 28964986 10.1016/j.mib.2017.09.004

[CR139] Pósfai G, Baldauf F, Erdei S, Pósfai J, Venetianer P, Kiss A (1984) Structure of the gene coding for the sequence-specific DNA-methyltransferase of the B. subtilis phage SPR. Nucleic Acids Res 12(23):9039–9049 6096817 10.1093/nar/12.23.9039 PMC320436

[CR140] Pritikin WB, Reiter H (1969) Abortive infection of Bacillus subtilis bacteriophage PBS1 in the presence of actinomycin D. J Virol 3(6):578–585 4978941 10.1128/jvi.3.6.578-585.1969 PMC375815

[CR141] Rao VB, Fokine A, Fang Q, Shao Q (2023) Bacteriophage T4 head: structure, assembly, and genome packaging. Viruses 15(2):527 36851741 10.3390/v15020527 PMC9958956

[CR142] Reeves PR, Cunneen MM, Liu B, Wang L (2013) Genetics and evolution of the Salmonella galactose-initiated set of O antigens. PLoS ONE, 8(7), e69306 10.1371/journal.pone.0069306 PMC3715488 23874940

[CR143] Rezaei M, Jalali A, Al-Azzawi DHS (2025) Engineered Bacteriophages: Advances in Phage Genome Redesign Strategies for Therapeutic and Environmental Applications. Protein \& Peptide Letters 10.2174/0109298665372719250616085616 40611420

[CR144] Riede I (1987) Receptor specificity of the short tail fibres (gp12) of T-even type Escherichia coli phages. Mol Gen Genet MGG 206:110–115 3553859 10.1007/BF00326544

[CR146] Roberts RJ, Belfort M, Bestor T, Bhagwat AS, Bickle TA, Bitinaite J, Blumenthal RM, Degtyarev SK, Dryden DTF, Dybvig K (2003) & others. A nomenclature for restriction enzymes, DNA methyltransferases, homing endonucleases and their genes. *Nucleic Acids Research*, *31*(7), 1805–1812 10.1093/nar/gkg274 PMC152790 12654995

[CR147] Roberts RJ, Vincze T, Posfai J, Macelis D (2015) REBASE—a database for DNA restriction and modification: enzymes, genes and genomes. Nucleic Acids Res 43(D1):D298–D299 25378308 10.1093/nar/gku1046 PMC4383893

[CR145] Roberts CG, Fishman CB, Zhang Z, Banh DV, Patel DJ, Marraffini LA (2025) Bacterial TIR-based immune systems sense phage capsids to initiate defense. Nat Microbiol, 1–11 10.1038/s41564-025-02150-0 PMC12578639 41136732

[CR148] Romeyer Dherbey J, Parab L, Gallie J, Bertels F (2023) Stepwise evolution of E. coli C and $Φ$X174 reveals unexpected lipopolysaccharide (LPS) diversity. Mol Biol Evol 40(7):msad154 37399035 10.1093/molbev/msad154 PMC10368449

[CR149] Roos WH, Ivanovska IL, Evilevitch A, Wuite GJL (2007) Viral capsids: mechanical characteristics, genome packaging and delivery mechanisms. Cell Mol Life Sci 64:1484–1497 17440680 10.1007/s00018-007-6451-1 PMC2771126

[CR150] Rostøl JT, Marraffini L (2019) (Ph) ighting phages: how bacteria resist their parasites. Cell Host \& Microbe 25(2):184–194 30763533 10.1016/j.chom.2019.01.009 PMC6383810

[CR151] Safari F, Sharifi M, Farajnia S, Akbari B, Baba Ahmadi K, Negahdaripour M, M., Ghasemi Y (2020) The interaction of phages and bacteria: the co-evolutionary arms race. Crit Rev Biotechnol 40(2):119–137 31793351 10.1080/07388551.2019.1674774

[CR152] Sahu R, Singh AK, Kumar A, Singh K, Kumar P (2023) Bacteriophages concept and applications: a review on phage therapy. Curr Pharm Biotechnol 24(10):1245–1264 36336808 10.2174/1389201024666221104142457

[CR153] Samson JE, Magadán AH, Sabri M, Moineau S (2013) Revenge of the phages: defeating bacterial defences. Nat Rev Microbiol 11(10):675–687 23979432 10.1038/nrmicro3096

[CR154] Scaltriti E, Launay H, Genois M-M, Bron P, Rivetti C, Grolli S, Ploquin M, Campanacci V, Tegoni M, Cambillau C& others. (2011) Lactococcal phage p2 ORF35-Sak3 is an ATPase involved in DNA recombination and AbiK mechanism. Mol Microbiol 80(1):102–116 21276096 10.1111/j.1365-2958.2011.07561.x

[CR155] Scholl D, Adhya S, Merril C (2005) Escherichia coli K1’s capsule is a barrier to bacteriophage T7. Appl Environ Microbiol 71(8):4872–4874 16085886 10.1128/AEM.71.8.4872-4874.2005 PMC1183359

[CR156] Selvaraj C, Singh SK (2020) Phage protein interactions in the inhibition mechanism of bacterial cell. Biocommun Phages, 121–142

[CR157] Seniya SP, Jain V (2022) Decoding phage resistance by mpr and its role in survivability of Mycobacterium smegmatis. Nucleic Acids Res 50(12):6938–6952 35713559 10.1093/nar/gkac505 PMC9262609

[CR158] Shabbir MAB, Hao H, Shabbir MZ, Wu Q, Sattar A, Yuan Z (2016) Bacteria vs. bacteriophages: parallel evolution of immune arsenals. Front Microbiol 7:1292 27582740 10.3389/fmicb.2016.01292 PMC4987407

[CR159] Sharma G, Sharma S, Sharma P, Chandola D, Dang S, Gupta S, Gabrani R (2016) Escherichia coli biofilm: development and therapeutic strategies. J Appl Microbiol 121(2):309–319 26811181 10.1111/jam.13078

[CR160] Shi Y, Masic V, Mosaiab T, Rajaratman P, Hartley-Tassell L, Sorbello M, Goulart CC, Vasquez E, Mishra BP, Holt S (2024) & others. Structural characterization of macro domain–containing Thoeris antiphage defense systems. *Science Advances*, *10*(26), eadn3310 10.1126/sciadv.adn3310 PMC11204291 38924412

[CR161] Shuwen H, Kefeng D (2022) Intestinal phages interact with bacteria and are involved in human diseases. Gut Microbes 14(1):2113717 36037202 10.1080/19490976.2022.2113717 PMC9427043

[CR162] Skliros D, Droubogiannis S, Kalloniati C, Katharios P, Flemetakis E (2023) Perturbation of Quorum Sensing after the Acquisition of Bacteriophage Resistance Could Contribute to Novel Traits in Vibrio alginolyticus. Microorganisms 11(9):2273 37764117 10.3390/microorganisms11092273 PMC10535087

[CR163] Skutel M, Andriianov A, Zavialova M, Kirsanova M, Shodunke O, Zorin E, Golovshchinskii A, Severinov K, Isaev A (2023) T5-like phage BF23 evades host-mediated DNA restriction and methylation. Microlife 4:uqad044 38025991 10.1093/femsml/uqad044 PMC10644984

[CR164] Smith WPJ, Wucher BR, Nadell CD, Foster KR (2023) Bacterial defences: mechanisms, evolution and antimicrobial resistance. Nat Rev Microbiol 21(8):519–534 37095190 10.1038/s41579-023-00877-3

[CR165] Sørensen PE, Baig S, Stegger M, Ingmer H, Garmyn A, Butaye P (2021) Spontaneous phage resistance in avian pathogenic Escherichia coli. Front Microbiol 12:782757 34966369 10.3389/fmicb.2021.782757 PMC8711792

[CR166] Stone E, Campbell K, Grant I, McAuliffe O (2019) Understanding and exploiting phage–host interactions. Viruses 11(6):567 31216787 10.3390/v11060567 PMC6630733

[CR167] Studier FW, Movva NR (1976) SAMase gene of bacteriophage T3 is responsible for overcoming host restriction. J Virol 19(1):136–145 781304 10.1128/jvi.19.1.136-145.1976 PMC354840

[CR168] Sumrall ET, Keller AP, Shen Y, Loessner MJ (2020) Structure and function of Listeria teichoic acids and their implications. Mol Microbiol 113(3):627–637 31972870 10.1111/mmi.14472

[CR169] Suttle CA (2007) Marine viruses—major players in the global ecosystem. Nat Rev Microbiol 5(10):801–812 17853907 10.1038/nrmicro1750

[CR170] Synefiaridou D, Veening J-W (2021) Harnessing CRISPR-Cas9 for genome editing in Streptococcus pneumoniae D39V. Appl Environ Microbiol 87(6):e02762–e02720 33397704 10.1128/AEM.02762-20 PMC8105017

[CR171] Taati Moghadam M, Amirmozafari N, Shariati A, Hallajzadeh M, Mirkalantari S, Khoshbayan A, Jazi M, F (2020) How phages overcome the challenges of drug resistant bacteria in clinical infections. Infect Drug Resist, 45–61 10.2147/IDR.S234353 PMC6954843 32021319

[CR173] Tal N, Morehouse BR, Millman A, Stokar-Avihail A, Avraham C, Fedorenko T, Yirmiya E, Herbst E, Brandis A, Mehlman T (2021) & others. Cyclic CMP and cyclic UMP mediate bacterial immunity against phages. *Cell*, *184*(23), 5728–5739 10.1016/j.cell.2021.09.031 PMC9070634 34644530

[CR172] Tal N, Millman A, Stokar-Avihail A, Fedorenko T, Leavitt A, Melamed S, Yirmiya E, Avraham C, Brandis A, Mehlman T& others (2022) Bacteria deplete deoxynucleotides to defend against bacteriophage infection. Nat Microbiol 7(8):1200–1209 35817891 10.1038/s41564-022-01158-0

[CR174] Tan D, Svenningsen S, Lo, Middelboe M (2015) Quorum sensing determines the choice of antiphage defense strategy in Vibrio anguillarum. MBio 6(3):10–1128 10.1128/mBio.00627-15 PMC4471561 26081633

[CR175] Teklemariam AD, Al-Hindi RR, Qadri I, Alharbi MG, Ramadan WS, Ayubu J, Al-Hejin AM, Hakim RF, Hakim FF, Hakim RF (2023) & others. The battle between bacteria and bacteriophages: a conundrum to their immune system. *Antibiotics*, *12*(2), 381 10.3390/antibiotics12020381 PMC9952470 36830292

[CR176] Vaitekenas A, Tai AS, Ramsay JP, Stick SM, Kicic A (2021) Pseudomonas aeruginosa resistance to bacteriophages and its prevention by strategic therapeutic cocktail formulation. Antibiotics 10(2):145 33540528 10.3390/antibiotics10020145 PMC7912912

[CR177] van Dalen R, Peschel A, van Sorge NM (2020) Wall teichoic acid in Staphylococcus aureus host interaction. Trends Microbiol 28(12):985–998 32540314 10.1016/j.tim.2020.05.017

[CR178] van den Berg DF, Costa AR, Esser JQ, Muralidharan A, van den Bossche H, Brouns SJJ (2025) Discovery of phage defense systems through component modularity networks. BioRxiv, 2009–2025

[CR179] van Houte S, Buckling A, Westra ER (2016) Evolutionary ecology of prokaryotic immune mechanisms. Microbiol Mol Biol Rev 80(3):745–763 27412881 10.1128/MMBR.00011-16 PMC4981670

[CR180] Vaysset H, Bernheim A (2025) The multifaceted roles of NAD + in bacterial immunity. Mol Cell 85(20):3743–3759 41106366 10.1016/j.molcel.2025.09.003

[CR181] Vinga I, Baptista C, Auzat I, Petipas I, Lurz R, Tavares P, Santos MA, São-José C (2012) Role of bacteriophage SPP1 tail spike protein gp21 on host cell receptor binding and trigger of phage DNA ejection. Mol Microbiol 83(2):289–303 22171743 10.1111/j.1365-2958.2011.07931.x

[CR182] Wang L, Jiang S, Deng Z, Dedon PC, Chen S (2019) DNA phosphorothioate modification—a new multi-functional epigenetic system in bacteria. FEMS Microbiol Rev 43(2):109–122 30289455 10.1093/femsre/fuy036 PMC6435447

[CR184] Wang S, Wan M, Huang R, Zhang Y, Xie Y, Wei Y, Ahmad M, Wu D, Hong Y, Deng Z& others (2021a) SspABCD-SspFGH constitutes a new type of DNA phosphorothioate-based bacterial defense system. Mbio 12(2):10–1128 10.1128/mBio.00613-21 PMC8092258 33906925

[CR186] Wang X, Loh B, Gordillo Altamirano F, Yu Y, Hua X, Leptihn S (2021b) Colistin-phage combinations decrease antibiotic resistance in Acinetobacter baumannii via changes in envelope architecture. Emerg Microbes \& Infections 10(1):2205–2219 10.1080/22221751.2021.2002671 PMC8648044 34736365

[CR187] Wang Y, Dai J, Wang X, Wang Y, Tang F (2022) Mechanisms of interactions between bacteria and bacteriophage mediate by quorum sensing systems. Appl Microbiol Biotechnol 106(7):2299–2310 35312824 10.1007/s00253-022-11866-6

[CR183] Wang M, Ji Q, Liu P, Liu Y (2023a) NAD+ depletion and defense in bacteria. Trends Microbiol 31(5):435–438 35803784 10.1016/j.tim.2022.06.002

[CR185] Wang X, Liu M, Yu C, Li J, Zhou X (2023b) Biofilm formation: mechanistic insights and therapeutic targets. Mol Biomed 4(1):49 38097907 10.1186/s43556-023-00164-w PMC10721784

[CR188] Wang Y, Fan H, Tong Y (2023c) Unveil the secret of the bacteria and phage arms race. Int J Mol Sci 24(5):4363 36901793 10.3390/ijms24054363 PMC10002423

[CR189] Warwick-Dugdale J, Buchholz HH, Allen MJ, Temperton B (2019) Host-hijacking and planktonic piracy: how phages command the microbial high seas. Virol J 16:1–13 30709355 10.1186/s12985-019-1120-1 PMC6359870

[CR190] Whitchurch CB, Tolker-Nielsen T, Ragas PC, Mattick JS (2002) Extracellular DNA required for bacterial biofilm formation. Science 295(5559):1487 11859186 10.1126/science.295.5559.1487

[CR191] Winstanley C, O’Brien S, Brockhurst MA (2016) Pseudomonas aeruginosa evolutionary adaptation and diversification in cystic fibrosis chronic lung infections. Trends Microbiol 24(5):327–337 26946977 10.1016/j.tim.2016.01.008 PMC4854172

[CR192] Wong JWH, Balskus EP (2025) Small molecules as modulators of phage–bacteria interactions. Curr Opin Chem Biol 84:102566 39736196 10.1016/j.cbpa.2024.102566

[CR193] Wu Y, Garushyants SK, van den Hurk A, Aparicio-Maldonado C, Kushwaha SK, King CM, Ou Y, Todeschini TC, Clokie MRJ, Millard AD (2024) & others. Bacterial defense systems exhibit synergistic anti-phage activity. *Cell Host \& Microbe*, *32*(4), 557–572 10.1016/j.chom.2024.01.015 PMC11009048 38402614

[CR194] Xia G, Maier L, Sanchez-Carballo P, Li M, Otto M, Holst O, Peschel A (2010) Glycosylation of wall teichoic acid in Staphylococcus aureus by TarM. J Biol Chem 285(18):13405–13415 20185825 10.1074/jbc.M109.096172 PMC2859500

[CR196] Xu X, Gu P (2024) Overview of Phage Defense Systems in Bacteria and Their Applications. Int J Mol Sci 25(24):13316 39769080 10.3390/ijms252413316 PMC11676413

[CR195] Xu L, Li J, Wu W, Wu X, Ren J (2024) Klebsiella pneumoniae capsular polysaccharide: Mechanism in regulation of synthesis, virulence, and pathogenicity. Virulence 15(1):2439509 39668724 10.1080/21505594.2024.2439509 PMC11649230

[CR197] Yin H, Li X, Wang X, Zhang C, Gao J, Yu G, He Q, Yang J, Liu X, Wei Y& others. (2024) Insights into the modulation of bacterial NADase activity by phage proteins. Nat Commun 15(1):2692 38538592 10.1038/s41467-024-47030-z PMC10973363

[CR199] Young RY (1992) Bacteriophage lysis: mechanism and regulation. Microbiol Rev 56(3):430–481 1406491 10.1128/mr.56.3.430-481.1992 PMC372879

[CR198] Young R (2005) Phage lysis. Phages: Their Role in Bacterial Pathogenesis and Biotechnology, 92–127

[CR201] Zhang M, Zhang T, Yu M, Chen Y-L, Jin M (2022) The life cycle transitions of temperate phages: regulating factors and potential ecological implications. Viruses, 14(9), 1904 10.3390/v14091904 PMC9502458 36146712

[CR200] Zhang H, You J, Li G, Rao Z, Zhang X (2025) From Natural Defense to Synthetic Application: Emerging Bacterial Anti-Phage Mechanisms and Their Potential in Industrial Fermentation. Fermentation 12(1):17

